# Comparative efficacy and safety of pulmonary surfactant delivery strategies in neonatal RDS: a network meta-analysis

**DOI:** 10.1186/s12890-024-03429-4

**Published:** 2024-12-30

**Authors:** Shiyue Liu, Yu Wang, Xingwang Zhu, Feifan Chen, Yuan Shi

**Affiliations:** 1 Department of Neonatology, Children’s Hospital of Chongqing Medical University, Chongqing, 400,014 China; 2 National Clinical Research Center for Child Health and Disorders, Hangzhou, 400,014 China; 3 Ministry of Education Key Laboratory of Child Development and Disorders, Chongqing, 400,014 China; 4 Chongqing Key Laboratory of Child Rare Diseases in Infection and Immunity, Chongqing, 400,014 China

**Keywords:** Neonatology, Neonatal Respiratory Distress Syndrome, Surfactant, Pulmonology, Critical Care, Nursing

## Abstract

**Purpose:**

To compare five pulmonary surfactant (PS) administration strategies for neonates with respiratory distress syndrome (RDS), including intubation-surfactant-extubation (InSurE), thin catheter administration, laryngeal mask airway (LMA), surfactant nebulization (SN), and usual care, with a particular emphasis on the comparison of the LMA and SN with other strategies.

**Methods:**

We conducted a systematic search of MEDLINE, EMBASE, PUBMED, and Cochrane CENTRAL databases up to November 2023. Two authors independently conducted data extraction, and assessed bias using the Cochrane Risk of Bias Tool. Frequency-based random-effects network meta-analyses were executed.

**Results:**

A total of 36 trials and 4035 infants were included in the analysis. LMA (OR: 0.20, 95%CI: 0.09 to 0.42) and Less Invasive Surfactant Administration (LISA) (OR: 0.17, 95%CI: 0.09 to 0.32) significantly reduced intubation rates compared to usual care. SN had a higher intubation rate compared to LISA (OR: 3.36, 95%CI: 1.46 to 7.71) and LMA (OR: 2.92, 95%CI: 1.10 to 7.71). LMA had a higher incidence of BPD compared to LISA (OR: 2.59, 95%CI: 1.21 to 5.54). SN ranked second to LISA in preventing BPD and death, but its efficacy decreased after excluding high-risk studies. SN and LMA had the lowest incidence of adverse events during administration.SN had the highest likelihood of secondary administration. Most results were rated as low or very low quality, with findings related to SN significantly impacted by high-risk trials.

**Conclusions:**

The thin catheter strategy minimized intubation risk and showed a better composite effect in reducing both mortality and BPD incidence. SN and LMA each showed safety and some clinical benefits in the subpopulations where they were studied, but their efficacy needs further validation through high-quality studies.

**Registration:**

This study was registered in PROSPERO (CRD42023463756).

**Supplementary Information:**

The online version contains supplementary material available at 10.1186/s12890-024-03429-4.

## Introduction

Neonatal Respiratory Distress Syndrome (RDS) is a major disease burden in premature newborns [1, 2], which is a pulmonary dysfunction triggered by insufficient pulmonary surfactant (PS) due to immature lung development. The incidence of neonatal RDS is increasing with the rising rate of preterm births and increased survival of extremely preterm infants [3, 4]. PS is a lipoprotein complex synthesized and secreted by alveolar type II epithelial cells, whose main function is to reduce alveolar surface tension and prevent alveoli from collapsing during expiration. The treatment of neonatal RDS aims to increase the level of PS and improve alveolar tension and gas exchange function. The introduction of lung surfactant replacement therapy has significantly improved the treatment of neonatal RDS [5].

Various methods of PS administration have been developed. Intubation-surfactant-extubation (InSurE) is used as the standard method of PS administration in the United States, with an emphasis on rapid extubation after intubation. However, data suggested that the incidence of bronchopulmonary dysplasia (BPD) in infants with RDS using this method was still as high as 40%, resulting in substantial healthcare costs [3, 6]. The InSurE method exposes infants to early tracheal intubation, and delayed extubation increases the duration of mechanical ventilation which is associated with an elevated risk of BPD. Many studies recommend that transitioning from invasive mechanical ventilation to early non-invasive continuous positive airway pressure (CPAP) significantly reduces the incidence of BPD [7]. However, this brought the critical dilemma to choose the InSurE for early drug administration or sustain CPAP. After that, the early administration of drugs through a catheter [8], called Less Invasive Surfactant Administration (LISA), which was endorsed by European guidelines [9, 10], made it possible to maintain CPAP. This approach not only reduced the mortality and the need for mechanical ventilation (MV) but also provided good prevention of BPD.

However, LISA and InSurE are invasive methods, as intratracheal surfactant administration requires airway manipulation, such as laryngoscopy, which can alter hemodynamics and increase risks like intraventricular hemorrhage (IVH). These methods also demand highly skilled practitioners and carry a risk of procedural failure. The pursuit of more non-invasive PS administration methods has always been the goal of neonatologists. Recently, there has been an increasing focus on the laryngeal mask airway (LMA) and surfactant nebulization (SN) methods. LMA avoids laryngoscopy and anesthesia, while SN offers a truly non-invasive approach, potentially providing greater comfort and less pain for the infant [11]. Nevertheless, the question persists whether a fully non-invasive method can elicit a positive therapeutic impact comparable to that of administering PS via an endotracheal tube or a catheter. Fortunately, a higher pulmonary drug deposition rate demonstrated by vibrating membrane nebulizers in recent research has restored confidence in SN [12].

In general, the various current strategies for the delivery of PS have both advantages and disadvantages. A comparable meta-analysis published in 2016 incorporated only a single article on SN and LMA [13]. Subsequently, a meta-analysis in 2021 addressed the same topic [14], yet the inadequate number of studies on SN and LMA persisted. And most of those included were observational studies. With an increasing number of recent studies on SN and LMA methods, it is imperative to conduct a comprehensive re-evaluation of research in this area to ascertain the clinical benefits of different interventions. In addition, the above two retrospective analyses paid more attention to the LISA method and highlighted clinical benefits like reduced BPD and intubation risk. Based on the above two studies, we have reduced our focus on LISA and paid more attention to exploring the potential benefits of SN and LMA in clinical practice.

Therefore, this review incorporated five strategies including InSurE, administration via thin catheter (LISA, Minimally Invasive Surfactant Therapy (MIST) and all extensions derived from these two methods), LMA, SN and usual care (postnatal non-invasive ventilation with surfactant administration through intubation if required). A frequentist network meta-analysis was employed to identify a more advantageous PS administration strategy for the average neonate with RDS, with a particular focus on the comparative safety and efficacy of the SN and LMA methods in relation to the other strategies.

## Methods

### Literature searches

We conducted a systematic search of the MEDLINE, EMBASE, Web of Science, and Cochrane library up to November 2023. In the search strategy, we used a combination of keywords related to various invasive and non-invasive treatment modalities (including LISA, MIST, LMA, InSurE, and nebulize) along with population and study type restrictions. Details of the search strategy are displayed in Supplementary file 1 (Supplementary Text 1). This study was registered in PROSPERO (CRD42023463756) before its commencement. We adhered to the PRISMA checklist in reporting this article (Appendix).

### Criteria for study inclusion and exclusion

We included RCTs that compared 2 or more of the predetermined 5 administration strategies (specific definitions of the strategies are provided in the Supplementary files (Supplementary Text 2)) and reported at least 1 event of the primary or secondary outcomes. Due to translation limitations and concerns about the accuracy of non-English studies, only English-language studies were included. To ensure robustness and reliability, we focused exclusively on randomized controlled trials (RCTs). For non-randomized controlled trials (non-RCTs), incomplete experiments, unreported results, studies that were not reported in English, and studies not primarily aimed at comparing the effectiveness of PS therapy for RDS, shall not be included.

Primary and Secondary Outcomes.

Our selected primary outcomes included the rate of intubation during the initial seven days of life, the prevalence of BPD, and mortality, which was chosen because BPD is the most important respiratory disease in preterm infants and the rate of intubation is closely related to BPD. Secondary outcomes included severe IVH, retinopathy of prematurity (ROP), neonatal necrotizing enterocolitis (NEC), patent ductus arteriosus (PDA), incidences of air leak, secondary dose administration (received two or more PS administrations), adverse events during surfactant administration, duration of mechanical ventilation, duration of oxygen support and length of hospital stay. Definitions and criteria for primary and secondary outcomes showed in supplementary files (Supplementary Text 3).

### Data extraction and risk of bias assessment

Data extraction was respectively performed by two authors utilizing a pre-designed form. The demographics and outcome data were extracted. We applied the Cochrane Risk of Bias Tool to evaluate potential biases [15]. Two researchers adhered to the Cochrane Handbook guidelines, independently evaluating each study concerning various outcome indicators. Any disagreements between reviewers during data extraction and the risk of bias were resolved through discussion, and in cases where consensus could not be reached, a third reviewer was consulted to provide an independent judgment (Supplementary Text 4).

### Data synthesis and analysis

During data analysis, we used the abbreviation LISA to represent all the transcatheter drug delivery strategies. Review Manager 5.3 and STATA 15.0 were used for statistical analyses. We performed a frequency-based network meta-analysis. Indirect comparisons were made through the common comparator, and the maximum likelihood estimation method was used to obtain effect values for mixed comparisons and to generate ranking probabilities for the corresponding measures [16]. Consistency between direct and indirect evidence was assessed using the node-splitting and loop-specific methods. The Surface Under the Cumulative Ranking Curve (SUCRA) was used to judge the relative merits and limitations [17]. A higher SUCRA value suggested a greater likelihood of superior or inferior performance compared to other methods.

### Quality of evidence assessment

The quality of evidence was assessed using the GRADE method for each comparative effect value and ranking probability for all primary and secondary outcomes [18, 19]. The GRADE evaluation of each network estimate focused on five dimensions: study limitations, indirectness, inconsistency, imprecision, and publication bias. Study limitations were downgraded if the contribution from low-risk-of-bias (ROB) evidence was insufficient. Imprecision triggered downgrading when effect estimates exceeded predefined limits, while indirectness was assessed based on the similarity of effect modifiers. Inconsistency led to downgrade if significant heterogeneity was found via prediction intervals and τ-square values. Publication bias was downgraded for comparisons with fewer than ten studies. The specific criteria for downgrading are displayed in the supplementary files (Supplementary Text 5).

### Sensitivity analysis

Subgroup analysis was not included in the protocol. However, due to the inconsistency of some results and baseline differences, we performed a post-hoc sensitivity analysis. We focused on clinically relevant variables that could potentially influence therapeutic outcomes. Specifically, gestational age was selected as a subgroup variable to account for differences in respiratory maturity. PEEP level was included because of its role in stabilizing alveolar recruitment and oxygenation. FiO₂ threshold was included because it reflects the severity of the disease and the different oxygen requirements of neonates. We also included nebulizer type, surfactant dose, and mode of ventilation as subgroup variables. Nebulizer type affects lung deposition efficiency, while different surfactant doses (100 mg/kg vs. 200 mg/kg) allow dose-dependent assessments. The choice of primary mode of ventilatory support (e.g. CPAP vs. NIPPV) introduces variability in ventilatory support, which may influence surfactant distribution and overall treatment outcomes. Additionally, we re-analyzed the results after excluding studies with a high risk of bias to minimize their influence on the results.

## Results

**Fig. 1:**
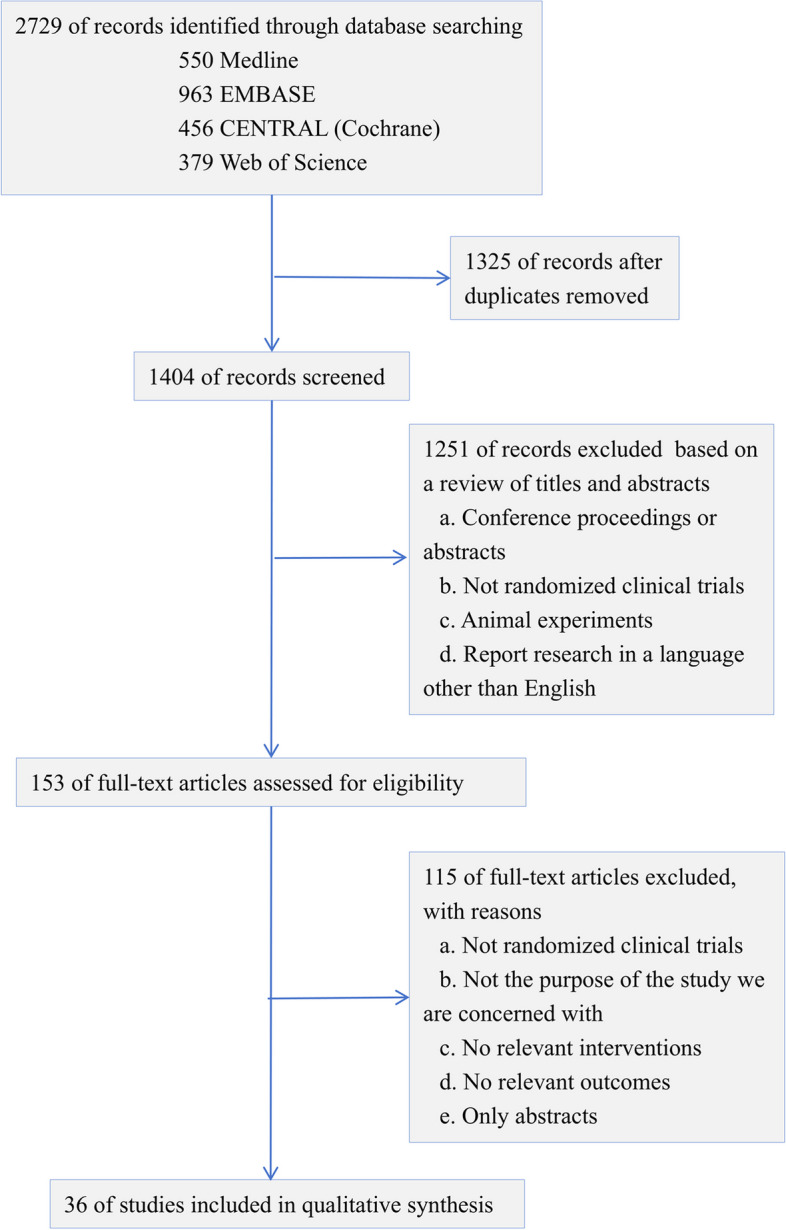
The flowchart of literature search and screening

**Fig. 2:**
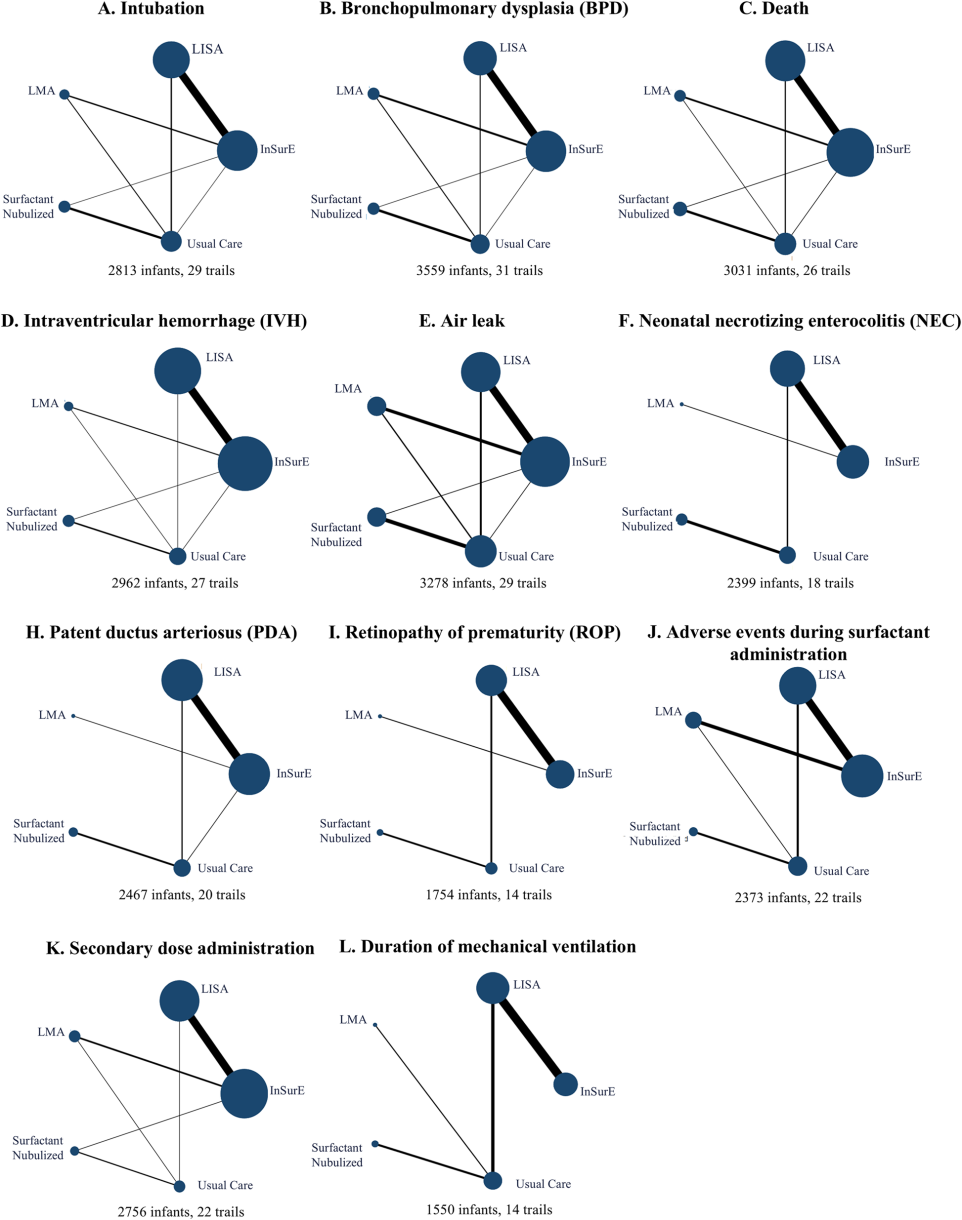
Network geometry for primary outcomes in network meta-analysis Each node depicted in the diagram represents a ventilation strategy, with its size directly correlating to the quantity of infants subjected to that specific ventilation approach. The interconnecting lines delineating the nodes signify direct comparisons between two strategies, with the thickness of each line proportionate to the number of trials directly comparing these respective ventilation strategies

A total of 2729 studies were retrieved and 35 full articles and 1 unpublished clinical trial were screened for inclusion (Figs. 1 and 2). 4035 cases of neonates aged 25–36 weeks (except one study) were included, of which 21.9% were recruited from Europe, 22.3% from North America, and 53.2% from Asia, with a mean gestational age of 31.10 (95% CI: 30.31–31.88) weeks. The average enrollment time after birth is less than 12 h, the fraction of inspired oxygen (FiO_2_) thresholds ranged from 0.22–0.6, and positive end-expiratory pressure (PEEP) ranged from 4–8 cm H_2_O. All baseline information and characteristics of the included studies are presented in Table 1 (Table 1).

**Table 1:** Baseline characteristics of included trials

Study Name	Year	Method of surfactant administration	Recruitment	No. of Infants	Multi/Single Centre	Gestational Age at Birth, Mean (SD)	Gestational Age at Birth, Median (IQR)/Range, w	Gestational Weight at Birth, Mean (SD), Median (IQR/Range), g	Infant Age at Enrollment	Single administration dose, mg/Kg	primary mode of respiratory support	FIO_2_ Threshold for Intervention
Mishra, A.,2023 [20]	2023	LISA	India	75	single‐center	31.41 (0.30)	28–36	1226 (176)	-	100	NIPPV	0.3
		InSurE		75		31.36 (0.69)	28–36	1262 (244)	-	100	NIPPV	0.3
Kaleem, A.,2023 [21]	2023	LISA	Pakistan	36	single‐center	-	26–34	-	< 6 h	200	nCPAP	0.3
		InSurE		36		-	27–34	-	< 6 h	200	nCPAP	0.3
Sabzehei, M. K.,2022 [22]	2022	LISA	Iran	56	single‐center	29.65 (3.02)	28–36	1530 (507)	-	200	nCPAP	0.4
		InSurE		56		30.58 (3.40)	28–36	1678 (543)	-	200	nCPAP	0.4
Anand, R,2022 [23]	2022	LISA	India	74	single‐center	30.48 (2.12)	25–34	1368 (341)	< 6 h	100	CPAP	0.3
		InSurE		76		30.06 (2.01)	25–34	1294 (328)	< 6 h	100	nCPAP	0.3
Pareek, P.,2021 [24]	2021	LISA	India	20	single‐center	31.36 (2.48)	28–36	1460 (580)	< 24 h	100	NIPPV	0.3 (< 30w) 0.4 (> 30w)
		InSurE		20		31.46 (2.40)	28–36	1500 (500)	< 24 h	100	NIPPV	
Akcay, N,2021 [25]	2021	LISA	Turkey	42	single‐center	-	29 (27-32)	1250 (1010–1605)	-	200	NIPPV	0.3 (< 26w) 0.4 (26-34w)
		InSurE		36		-	30 (28–32)	1200 (955–1767.5)	-	200	NIPPV	
Yang, G.,2020 [26]	2020	LISA	China	47	single‐center	33.70 (1.00)	32–36	2106 (315)	< 12 h	200	nCPAP	0.4
		InSurE		50		34.10 (1.30)	32–36	2219 (314)	< 12 h	200	nCPAP	0.4
Han, T.,2020 [27]	2020	LISA	China	151	multi-center	33.70 (1.00)	25–32	2106 (315)	< 6 h	100	nCPAP	0.4
		InSurE		147		34.10 (1.30)	25–32	2219 (314)	< 6 h	100	nCPAP	0.4
Gupta, B. K.,2020 [28]	2020	LISA	Indian	29	single‐center	30.07 (1.51)	28–34	1225 (281)	< 6 h	200	NIPPV	0.3
		InSurE		29		29.90 (1.67)	28–34	1222 (322)	< 6 h	200	NIPPV	0.3
Jena, Soumya R.,2019 [29]	2013–2017	LISA	India	175	multi-center	-	31 (29‐33)	1630 (1217‐2058)	< 6 h	135	nCPAP	0.3
		InSurE		175		-	31 (29‐33)	1683 (1316‐2041)	< 6 h	135	nCPAP	0.3
Boskabadi, H.,2019 [30]	2019	LISA	Iran	20	single‐center	29.10 (2.60)	< 32	1280(314)	-	200	nCPAP	0.4
		InSurE		20		28.20 (2.10)	< 32	1230(221)	-	200	nCPAP	0.4
Choupani, R.,2018 [31]	2016–2017	LISA	Iran	52	single‐center	32.90 (2.60)	-	1938 (555)	< 1 h	200	nCPAP	0.4
		InSurE		52		33.06 (2.30)	-	2067 (573)	< 1 h	200	nCPAP	0.4
Olivier, F.,2022 [32]	2022	LISA	Canada	24	multi-center	34.00 (1.40)	32–36	2157 (487)	< 24 h	100	nCPAP	0.35
		Usual care		21		33.90 (1.50)	32–36	2277 (658)	< 24 h	100	nCPAP/NIPPV	0.35
Mosayebi, Z.,2017 [33]	2017	LISA	Iran	27	single‐center	32.60 (1.10)	28–34	1792 (554)	-	200	NIPPV	0.4
		InSurE		26		31.90 (1.50)	28–34	1910 (433)	-	200	NIPPV	0.4
Mohammadizadeh M,2015 [34]	2015	LISA	Iran	19	multi-center	30.00 (2.00)	< 34	1289 (219)	< 1 h	200	nCPAP	0.3
		InSurE		19		31.00 (2.00)	< 34	1428 (272)	< 1 h	200	nCPAP	0.3
Kribs, Angela,2015 [35]	2009–2012	LISA	Germany	107	multi-center	25.30 (1.10)	23–27	711 (195)	< 2 h	100	nCPAP	0.3
		Usual care		104		25.20 (0.91)	23–27	674 (165)	< 2 h	100	nCPAP	0.3
Nayeri, F. S,2014 [36]	2014	InSurE	Iran	21	single‐center	31.00 (2.60)	< 35	1532 (539)	-	100	nCPAP	0.45
		Usual care		21		30.30 (2.87)	< 35	1485 (572)	-	100	nCPAP	0.45
Kanmaz, H. G.,2013 [37]	2010–2011	LISA	Turkey	100	single‐center	28.00 (2.00)	< 32	1093 (270)	< 2 h	100	nCPAP	0.4
		InSurE		100		28.30 (2.00)	< 32	1121 (270)	< 2 h	100	nCPAP	0.4
Halim, A.,2019 [38]	2019	LISA	Pakistan	50	single‐center	-	< 34	1300 (600)	< 12 h	100	nCPAP	0.4
		InSurE		50		-	< 34	1400 (400)	< 12 h	100	nCPAP	0.4
Goepel, Wolfgang,2011 [39]	2007–2010	LISA	Germany	108	multi-center	27.60 (0.80)	26–28	975 (244)	< 12 h	100	nCPAP	0.3
		Usual care		112		27.50 (0.80)	26–28	938 (205)	< 12 h	100	nCPAP	0.3
Kayvan Mirnia,2013 [40]	2010–2012	LISA	Iran	32	single‐center	30.08 (1.50)	< 32	1383 (58)	-	200	nCPAP	0.3
		InSurE		40		29.60 (2.50)	< 32	1490 (77)	-	200	nCPAP	0.3
Yingying Bao,2015 [41]	2012	LISA	China	47	single‐center	29.10 (1.50)	28–32	1034 (221)	< 2 h	200	nCPAP	0.3
		InSurE		43		29.30 (1.60)	28–32	1087 (198)	< 2 h	200	nCPAP	0.3
Gaertner VD,2023 [42]	2023	SN	Switzerland	18	single‐center	-	29.4 (29.0–31.1)	1080 (930–1430)	-	200	nCPAP	-
		Usual care		17		-	30.1 (27.0–31.1)	1140 (880–1360)	-	200	nCPAP	-
Sadeghnia, A. R.,2022 [43]	2019–2021	SN	Iran	25	single‐center	30.30 (1.04)	28–32	1440(316)	< 2 h	200	nCPAP	0.4
		InSurE		25		29.80 (1.31)	28–32	1363 (365)	< 2 h	200	nCPAP	0.4
Cummings JJ,2020 [44]	2020	SN	America	230	multi-center	33.20 (3.20)	28–41	2126 (828)	1-12 h	200	nCPAP	-
		Usual care		227		33.10 (3.10)	28–41	2081 (769)	1-12 h	200	nCPAP	-
Kaluarachchi, D. C.,2023 [45]	2023	SN	America	189	multi-center	-	28–36	1910 (1480–2400)	1-12 h	200	nCPAP	-
		Usual care		164		-	28–36	1900 (1530,2380)	1-12 h	200	nCPAP	-
Minocchieri S,2019 [46]	2010–2012	SN	Australia	32	single‐center	31.40 (1.40)	29–33	1562 (399)	< 4 h	200	nCPAP	0.22–0.3
		Usual care		32		31.40 (1.40)	29–33	1645 (409)	< 4 h	200	nCPAP	0.22–0.3
Dani C,2022 [47]	2022	SN	Europe	83	multi-center	30.65 (1.30)	28–33	1399(383)	1-12 h	200(400)	nCPAP	0.25–0.4
		Usual care		43		30.60 (1.40)	28–33	1450 (346)	1-12 h	200(400)	nCPAP	0.25–0.4
Berggren E,2000 [48]	2000	SN	Sweden	16	multi-center	-	31 (28–33) ^a^	1620 (1015–2370)	2-36 h	480	nCPAP	0.4
		Usual care		16		-	31 (27–34) ^a^	1603 (755–2855)	2-36 h	480	nCPAP	0.4
NCT02074059, [49]	2014–2015	SN	America	40	multi-center	-	29–34	-	< 21 h	25–150	nCPAP	-
		Usual care		40		-	29–34	-	< 21 h	25–150	nCPAP	-
Gallup, J. A.,2023 [50]	2014–2020	LMA	America	51	single‐center	31.70 (2.10)	27–36	1926 (555)	< 48 h	105	nCPAP	0.3–0.6
		InSurE		42		31.60 (2.60)	27–36	1848 (676)	< 48 h	105	nCPAP	0.3–0.6
Amini, E.,2019 [51]	2014–2015	LMA	Iran	30	single‐center	32.60 (2.30)	< 37	1970 (520)	< 2 h	200	nCPAP	0.3–0.6
		InSurE		30		32.40 (2.30)	< 37	1850 (496)	< 2 h	200	nCPAP	0.3–0.6
Roberts, K. D.,2017 [52]	2017	LMA	America	50	single‐center	32.71 (1.86)	28–36	1968 (506)	< 36 h	200	nCPAP	0.3–0.4
		Usual care		53		32.86 (1.86)	28- < 36	1995 (483)	< 36 h	200	nCPAP	0.3–0.4
Gharehbaghi, M.,2018 [53]	2018	LMA	Iran	25	single‐center	32.88 (1.32)	33–37	2078 (669)	-	100	nCPAP	-
		InSurE		25		33.76 (2.12)	33–37	2198 (669)	-	100	nCPAP	-
Pinheiro, J. M. B.,2015 [54]	2010–2012	LMA	America	30	single‐center	-	28–35 (< 36)	2118 (1150–3984)	4-48 h	105	nCPAP/NIPPV	0.3–0.6
		InSurE		30		-	28–35 (< 36)	1945 (1015–3700)	4-48 h	105	nCPAP/NIPPV	0.3–0.6
Attridge, J. T.,2013 [55]	2013	LMA	America	13	single‐center	-	33.7 (31.0–34.9)	2001 (1670–2820)	< 72 h	105	nCPAP	0.3–0.6
		Usual care		13		-	32.0 (31.4–35.0)	2130 (1799–2570)	< 72 h	105	nCPAP	0.3–0.6

*Abbreviations:* *FIO*_2_ Fraction of inspired oxygen, *InSurE* intubation-surfactant-extubation, *LISA* less invasive surfactant administration (including all methods of PS administration via catheter), *SN* surfactant nebulised, *LMA* laryngeal mask airway, *nCPAP* Nasal continuous positive airway pressure, *NIPPV* nasal intermittent positive pressure ventilation, *PEEP* Positive End-Expiratory Pressure, *IQR* interquartile range, *SD* standard deviation

^a^: Parentheses with ^a^ represent full range rather than interquartile range

### Risk of bias

One experiment grouped based on odd or even file numbers, was deemed high risk. Of the eight trials with SN intervention, three were funded by pharmaceutical companies and provided the drugs and nebuliser, which poses a high risk of bias. However, all three studies had an independent third party to monitor or audit the data to mitigate the influence of the funder. In two studies, ethical or informed consent was not clearly reported, and there may have been selective bias. Six studies terminated recruitment early due to COVID-19, funding issues, and recruitment challenges, potentially introducing bias. The remaining studies had either low or unknown risk in their results (Supplementary Table 1).

### Primary outcomes

**Fig. 3:**
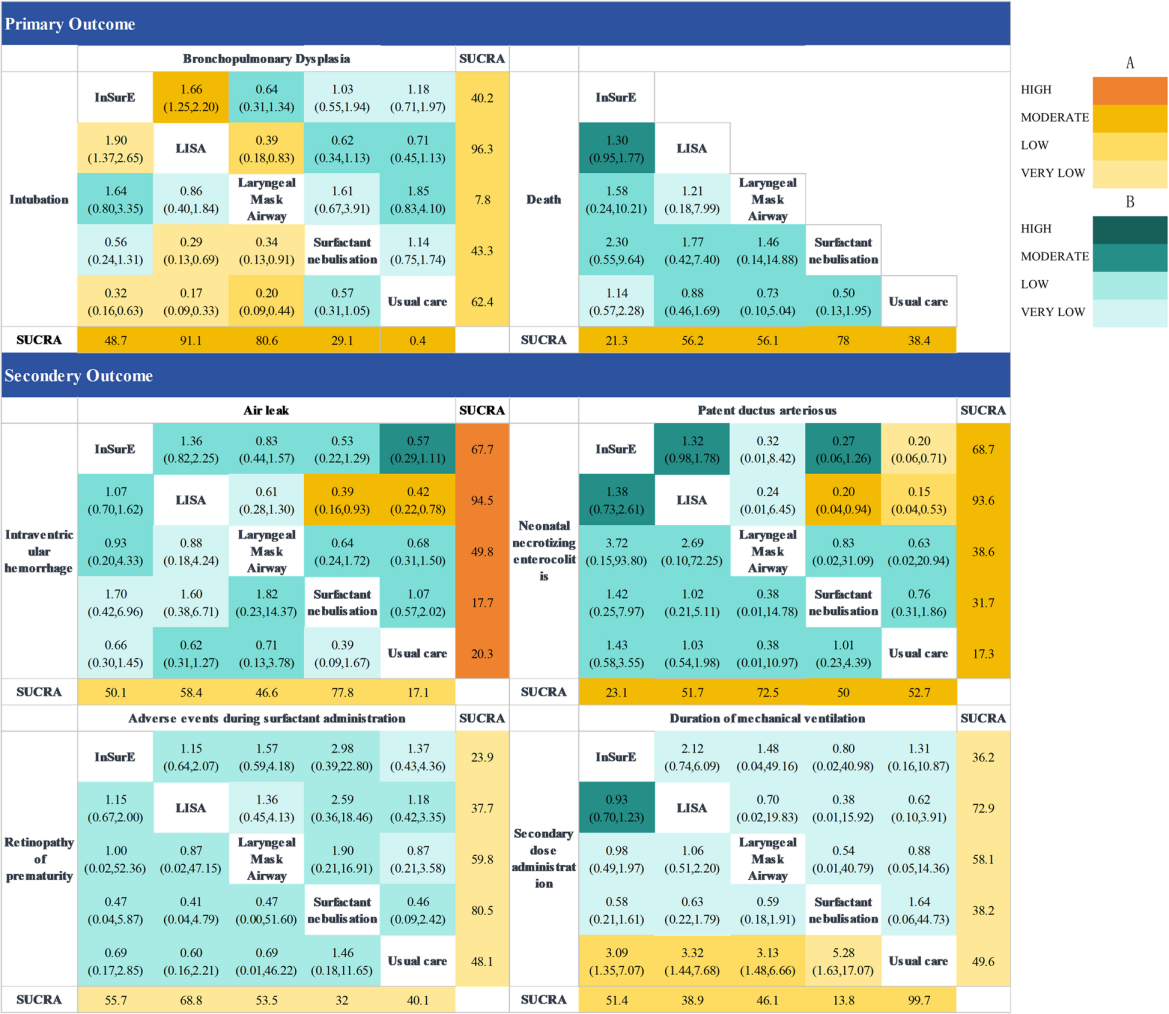
Effect sizes of network meta-analysis and associated quality of evidence. **A **represents statistically significant results. **B **represents results that are not statistically significant. SUCRA: Surface Under the Cumulative Ranking HIGH, high quality; MODERATE, moderate quality; LOW, low quality; VERY LOW, very low quality

**Fig. 4:**
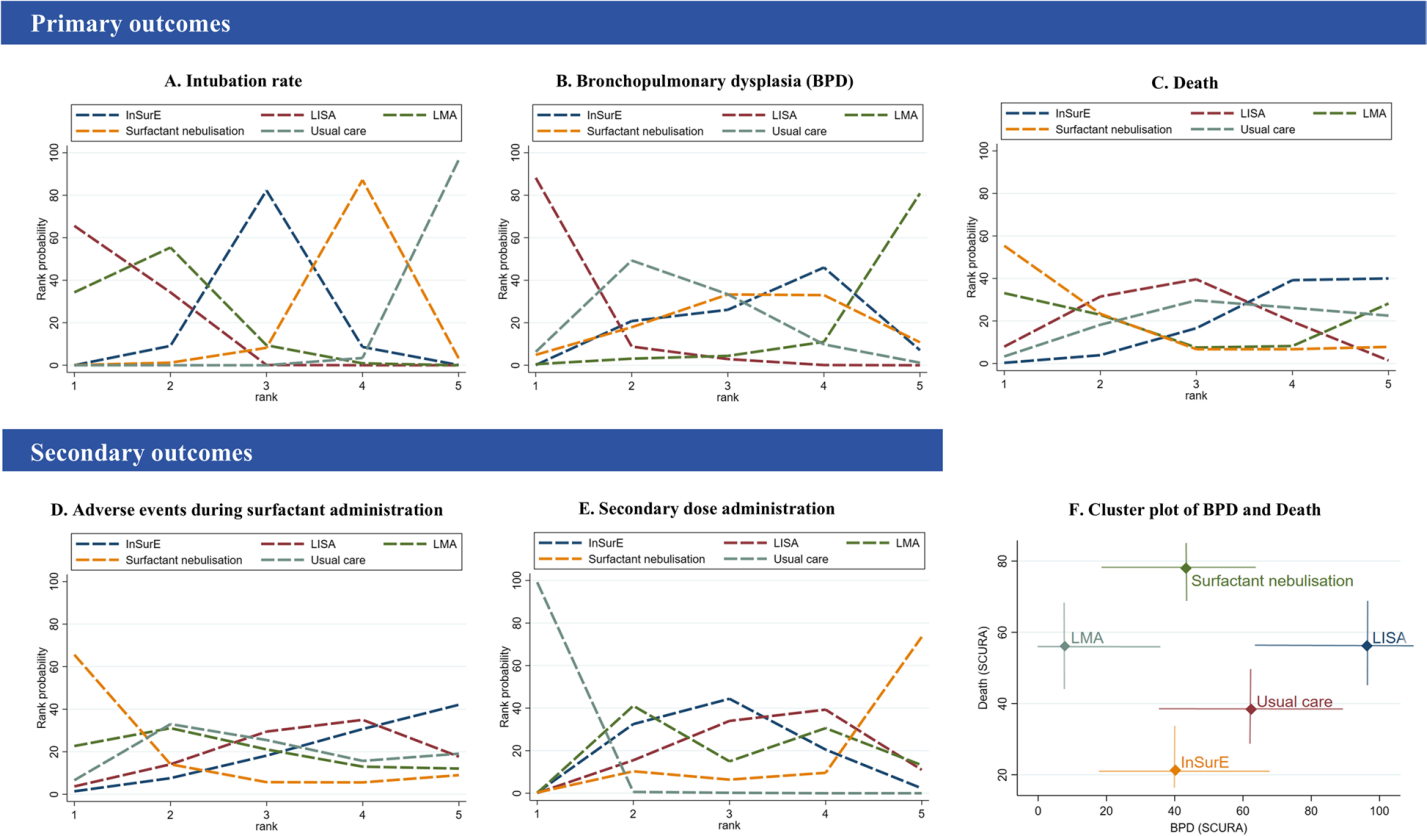
The ranking probability of strategies and the SUCRA value in the network meta-analysis of surfactant administration. **A**, **B**, **C**, **D** and **E** represented the ranking probability plots for the primary outcomes (intubation, BPD and death) and secondary outcomes (adverse drug events, secondary drug administration). **F** showed the cluster plot of the two primary outcomes of BPD and death, with the horizontal and vertical coordinates representing the SUCRA values for each intervention across the different outcomes

Compared to usual care, this study found that LMA (OR: 0.20, 95% CI: 0.09–0.42), LISA (OR: 0.17, 95% CI: 0.09–0.32), and InSurE (OR: 0.32, 95% CI: 0.17–0.62) significantly reduced neonatal tracheal intubation within seven days after birth. There was no statistically significant difference in intubation rates between LMA and LISA, as well as between LMA and InSurE. SN had a higher intubation rate compared to LISA (OR: 3.36, 95% CI: 1.46–7.71) and LMA (OR: 2.92, 95% CI: 1.10–7.71) (Fig. 3). The probability ranking plot (Fig. 4) indicated that catheter-based administration had the lowest likelihood of intubation, followed closely by LMA, with SN having the highest probability of intubation rates.

BPD prevalence was higher with LMA administration compared to LISA (OR: 2.59, 95% CI: 1.21–5.54). Differences in BPD incidence between SN and LISA, as well as SN and InSurE, were not statistically significant. Probability ranking plots identified LISA as having the lowest likelihood of BPD, and SN was second only to LISA in its composite effect of simultaneously reducing BPD and mortality. (Fig. 4F).

### Secondary outcomes

The SUCRA value (Fig. 3) indicated that both SN and LMA strategies had a higher likelihood of fewer adverse events during administration, but head-to-head comparisons did not show statistically significant differences in effect size. InSurE, LISA, LMA, and SN had a higher incidence of secondary dosing compared to usual care. Probability ranking plots (Figs. 3 and 4) revealed that SN had the highest likelihood of necessitating secondary drug administration among all interventions. In terms of other secondary outcomes (Supplementary Fig. 1), LMA had the lowest probability of NEC. The ranking probabilities for reducing the risk of NEC were similar for LISA, SN and UC, but all were superior to the InSurE method. LISA did not exhibit a distinctive advantage in reducing the risk of NEC. IVH was least likely with SN, but it's noteworthy that pneumothorax (OR: 2.58, 95% CI: 1.08–6.17) and PDA (OR: 4.95, 95% CI: 1.07–22.93) (Fig. 3) were more likely with SN compared to LISA. Among the interventions, LISA demonstrated the highest probability of preventing pneumothorax and PDA, followed by InSurE. LMA also exhibited a greater likelihood of preventing pneumothorax and PDA than usual care. LISA exhibited the highest probability of preventing mechanical ventilation, followed by LMA, both of which were superior to usual care. Pre-specified outcomes, including duration of oxygen support and length of hospitalization, were dropped as secondary outcomes due to insufficient sample size and significant differences in heterogeneity.

### Quality of evidence assessment

Except for InSurE and LISA comparisons, none of the six head-to-head comparisons included over 10 original studies (Table 1). Consequently, we downgraded the quality of all comparisons, excluding InSurE and LISA, by one level due to publication bias (Supplementary Fig. 2) stemming from the limited number of original studies available. Additionally, to address the high risk of bias in intubation, BPD, and secondary administration outcomes, a uniform downgrade was applied to the quality of evidence across all comparisons and probability plots involving these three outcomes by one level. This cautious downgrading was necessary to acknowledge the potential impact of bias on the overall robustness of the evidence. The results of the quality of evidence assessment are shown in Fig. 4 (Fig. 4, Supplementary Table 2).

### Sensitivity and subgroup analyses

The loop-specific and side-splitting approaches revealed inconsistencies in the analyses of intubation, IVH, and second-dose administration (Supplementary Table 4&5). After standardizing PEEP levels, respiratory support modes, and nebulizer types, sensitivity analysis showed no inconsistencies in intubation outcomes. Similarly, inconsistencies in the second-dose administration were eliminated after excluding high-risk bias studies and standardizing PEEP levels, as well as in gestational age subgroup analyses. Following these adjustments, the results for intubation and second-dose administration remained unchanged. The IVH inconsistency originated from Sadeghnia et al.'s study. Their study's removal shifted the probability ranking of SN from first to last. Aside from dose–effect subgroup analyses, the SUCRA rankings remained stable in other sensitivity analysis, though most odds ratios for comparisons became non-significant. Moreover, excluding industry-sponsored studies reduced the likelihood of SN preventing BPD and death (Supplementary Table 3).

## Discussion

The study systematically evaluated the efficacy and safety of five PS delivery strategies for the treatment of RDS, including InSurE, LISA, LMA, SN and usual care. Among these strategies, the thin catheter strategy offers greater clinical advantages for the average neonate with RDS. It has the greatest potential to prevent intubation, BPD, and mortality and demonstrates the highest likelihood of minimizing complications like pneumothorax, PDA, and ROP, while having the lowest likelihood of requiring a second dose. Unlike the prior investigations conducted by Isayama T et al. [13] and Bellos I et al. [14], we focused on the potential benefits of SN and LMA in clinical practice and whether these strategies show comparable or superior therapeutic efficacy to LISA or InSurE. Fortunately, the final results demonstrated that LMA is non-inferior to LISA and superior to InSurE in reducing intubation rates. SN ranks second to LISA in its composite effect of simultaneously reducing BPD and mortality but shows diminished effectiveness when high-risk studies are excluded. Both SN and LMA have better safety compared to the other administration methods. However, compared with LISA and InSurE, LMA was associated with a higher likelihood of BPD, and SN showed no advantages in reducing intubation rates.

Transcatheter drug delivery has advantages over other delivery strategies. The conclusions of the analyses of the primary outcomes, including intubation, BPD and mortality, are consistent with those of a large number of previous clinical trials and meta-analyses. It avoids exposure to mechanical ventilation during administration, reduces laryngeal and vocal cord damage, maintains non-invasive respiratory support, and achieves uniform distribution of PS under an autonomous respiratory drive [20, 35, 39]. Nevertheless, it is essential to acknowledge that transcatheter drug delivery remains an invasive procedure for infants, and we cannot avoid the use of laryngoscopy. It also requires a high level of operator skill and carries an inherent risk of drug delivery failure. The results of our study support partial considerations, showing that relatively non-invasive measures such as SN and LMA may perform better in preventing adverse events during administration than thin-catheter administration. Moreover, some researchers believed that there were still some limitations in promoting the thin catheter strategy [10, 56, 57], such as the lack of consensus on whether to sedate and which sedative drugs to administer, the lack of standardized methods of assisted respiratory support and the pressure of the oxygen to be delivered during drug administration, not harmonized clinical decision thresholds for surfactant administration (e.g., thresholds for FIO_2_ and PEEP) and the existence of doubts about the smooth transmission of CPAP pressure during catheter drug administration [58]. However, in light of prevailing clinical evidence, transcatheter administration still emerges as particularly advantageous in tertiary neonatal centers equipped with comprehensive facilities.

Based on the current research and our findings, the use of LMA is a promising therapeutic strategy. This approach avoids the need for laryngoscopy and preoperative medication, while providing some improvement in decreasing intubation rates and mortality [59]. Furthermore, LMA administration exhibits a diminished likelihood of adverse events, including reflux, vomiting, and bradycardia during the administration process. Jacqueline A. Gallup et al. also identified that LMA was noninferior to administration via endotracheal tube and it decreased early failures [50]. However, the conclusions apply only to larger gestational-age infants, as laryngeal mask sizes for small preterm infants are unavailable. Our study also identified that LMA had the highest incidence of BPD among the evaluated administration strategies, a finding consistent across subgroups. This is unexpected, as previous studies classified the laryngeal mask as a supraglottic device with less airway stimulation than intubation. Two recent meta-analyses also reported a higher BPD incidence with LMA [59, 60], but these results were not statistically significant. BPD incidence is critical in evaluating RDS management, and if LMA cannot effectively reduce BPD, more cautious clinical decision-making is warranted. However, due to the small number of included trials on LMA, the current evidence is mainly based on indirect comparisons. Further studies are essential to validate this conclusion. Moreover, increased gastric leakage seems to be inevitable [52], as shown by many clinical studies. Further research may be warranted to develop products that can be adapted to smaller newborns and to better determine the correct placement of laryngeal masks. Anticipated benefits of these endeavors include the broader adoption of LMA. In general, the LMA strategy is poised to confer advantages, especially in neonates of advanced gestational age and within resource-constrained settings characterized by lower intubation requirements and limited proficiency in PS management [61].

The development of SN administration as a non-invasive drug delivery method has garnered significant interest. In our results, nebulizers have shown efficacy in reducing BPD and mortality related to neonatal RDS. However, when excluding industry-sponsored trials, the benefit of SN in preventing the combined outcome of BPD and death was reduced, indicating that such trials may have overestimated SN’s clinical effect. Furthermore, the intubation rate for SN was higher compared to other methods, which may have a compensatory effect on clinical efficacy.

Another consideration is that SN exhibits the highest likelihood of secondary administration compared to other interventions, suggesting a potential inefficiency relative to alternative delivery strategies. The effectiveness of SN depends on multiple factors, including the choice of nebulizer [44, 48, 62], the parameters of non-invasive ventilation [7, 63], the age and timing of administration, and the rate of drug deposition in the lungs [64–66]. Vincent D Gaertner et al. reported in their small study that there may be no clinically meaningful effect of prophylactic SN on lung ventilation [42]. In vitro studies have indicated that recently developed vibrating membrane nebulizers can achieve lung deposition rates of up to 14% [64], and when combined with non-invasive positive pressure ventilation (NIPPV), the deposition rates can reach up to 20% [63] presenting a substantial improvement compared to earlier jet nebulizers. Despite the relatively lower lung deposition rates in comparison to tracheal drip, animal studies corroborated that nebulizing the same nominal dose of PS can produce pulmonary responses comparable to endotracheal drip [62, 67]. This may indicate that augmenting the clinical dose could serve as a compensatory measure for the reduced efficacy of drug delivery [44]. But how to make SN delivery more efficient still deserves further research.

Our results also showed the lowest likelihood of adverse events during administration with the SN strategy. It reflects that SN could avoid the acute effect of laryngoscopy and the acute airway fluid load associated with surfactant instillation while maintaining stable hemodynamics. Beena G. Sood also indicated that SN exerts a less pronounced influence on cerebral blood flow in comparison to LISA and InSurE [68]. However, consistent with previous studies, the increased likelihood of pneumothorax with SN usage remains a concern [42]. To mitigate excessive alveolar pressure, careful consideration of appropriate positive airway pressure and peak inspiratory pressure is imperative.

### Limitations

Our study has several limitations. First, we lacked clinical data on low gestational age. The limitation of gestational age is critical in RDS treatment research, as different ages may reflect different levels of distress. We conducted subgroup analyses by gestational age, dividing it into 28–32 weeks and > 32 weeks, but studies involving extremely preterm infants were only found in the comparison between LISA and UC, preventing comparisons between different interventions for extremely preterm infants with RDS. Additionally, we could not determine the type of ventilation used in intubated infants. Using oscillation and strict airway pressure control might have prevented some cases of BPD in these patients. It is also possible that intubated infants had more severe respiratory distress syndrome than non-intubated infants, which could have affected the results. Furthermore, more RCTs were conducted in low- and middle-income countries than high-income countries, where standards of care may differ significantly. However, this was somewhat mitigated by the inclusion of a comparable number of infants from both settings. Lastly, significant variability in the volume of evidence across comparisons may introduce bias into the network structure. The exclusion of non-English reports and non-RCT studies may also give rise to selection bias.

Baseline differences among the included studies may have contributed to variability in the pooled effect sizes. Fortunately, sensitivity analyses accounting for these differences yielded results consistent with the original findings, indicating the robustness of the primary outcomes. However, each study is optimally designed for its target population, so intransitivity must be considered when interpreting the results. Subgroup analysis of dose effects showed considerable deviations, which are likely due to the exclusion of studies with higher PS doses in the SN group, while the PS deposition rate in the SN group with an equivalent dose was comparatively lower. Additionally, most of our results were of low or very low quality, with outcomes related to atomized PS being more influenced by high-risk studies. Therefore, caution is advised when generalizing conclusions or making guideline recommendations.

## Conclusion

The thin catheter strategy was linked to the lowest intubation risk and a better composite effect in simultaneously reducing mortality and BPD incidence. Based on current evidence, surfactant administration via a thin catheter might have greater clinical advantages over other measures, though certain limitations continue to hinder its broader adoption. SN and LMA each showed safety and some clinical benefits in the subpopulations where they were studied, but their efficacy needs further validation through high-quality studies.

Future research could focus on whether to use preoperative sedation before catheter insertion and the long-term impact of neurodevelopmental outcomes for transcatheter administration. For SN and LMA, attempts could be made to expand the study population by supplementing studies on infants with lower gestational age and moderate-to-severe RDS. Additionally, enhancing the pulmonary deposition and delivery efficiency of nebulized surfactant remains a crucial area for future study. Strategies to improve deposition might include optimizing nebulizer type, adjusting flow rates, refining SN interfaces, comparing different surfactant concentrations, etc. Building on improved deposition efficiency, future studies could also compare nebulized PS with thin catheter administration like LISA and identify neonatal subgroups most likely to benefit from nebulized PS, such as those with mild RDS or those who cannot tolerate intubation.

## Supplementary Information

Supplementary Material 1: Supplementary Text 1. Search strategies. Supplementary Text 2. Criteria for Study Inclusion and Exclusion. Supplementary Text 3. Definitions and criteria for primary and secondary outcomes. Supplementary Text 4. Risk of bias of included trials. Supplementary Text 5. Quality of evidence assessment. Supplementary Table 1. Risk of bias of included trials. Supplementary Table 2. The results of the quality of evidence assessment. Supplementary Table 3. Sensitivity Analysis Results of SUCRA for Probability Ranking Plot and Odds Ratios for Pairwise Comparisons. Supplementary Table 4. Results of Loop-specific approach. Supplementary Table 5. Results of Side-splitting approach. Supplementary Fig 1. Plots of the surface under the cumulative ranking curves for all interventions. Supplementary Fig 2. Comparison-adjusted funnel plot with pseudo 95% confidence limits.

Supplementary Material 2. Appendix 1: Changes of the final review protocol from the original one in PROSPERO and reasons.

Supplementary Material 3. Appendix 2: PRISMA checklist of the network meta-analysis.

## Data Availability

The data analyzed during the current study are available from the corresponding author upon reasonable request. All data generated during this study are included in this published article and its supplementary information files.

## Abbreviations

PS: Pulmonary surfactant
RDS: Respiratory distress syndrome
RCTs: Randomized controlled trials
LISA: Less Invasive Surfactant Administration
BPD: Bronchopulmonary Dysplasia
SN: Surfactant Nebulization
LMA: Laryngeal Mask Airway
SRT: Surfactant Replacement Therapy
InSurE: Intubation-Surfactant-Extubation
CPAP: Continuous positive airway pressure
MIST: Minimally invasive surfactant therapy
PRISMA: Preferred Reporting Items for Systematic Reviews and Meta-Analyses
non-RCTs: Non-randomized controlled trials
IVH: Intraventricular hemorrhage
ROP: Retinopathy of prematurity
NEC: Neonatal necrotizing enterocolitis
PDA: Patent ductus arteriosus
SUCRA: Surface Under the Cumulative Ranking Curve
FiO_2_: The fraction of inspired oxygen
PEEP: Positive end-expiratory pressure
NIPPV: Nasal intermittent positive pressure ventilation (NIPPV)
