# Supplementary material for: Comparative efficacy and safety of pulmonary surfactant delivery strategies in neonatal RDS: a network meta-analysis
Source: BMC Pulm Med. 2024 Dec 30;24:637. doi: 10.1186/s12890-024-03429-4 (PMC11687079; doi:10.1186/s12890-024-03429-4)
Supplement: Supplementary file 1 — Supplementary Material 1: Supplementary Text 1. Search strategies. Supplementary Text 2. Criteria for Study Inclusion and Exclusion. Supplementary Text 3. Definitions and criteria for primary and secondary outcomes. Supplementary Text 4. Risk of bias of included trials. Supplementary Text 5. Quality of evidence assessment. Supplementary Table 1. Risk of bias of included trials. Supplementary Table 2. The results of the quality of evidence assessment. Supplementary Table 3. Sensitivity Analysis Results of SUCRA for Probability Ranking Plot and Odds Ratios for Pairwise Comparisons. Supplementary Table 4. Results of Loop-specific approach. Supplementary Table 5. Results of Side-splitting approach. Supplementary Fig 1. Plots of the surface under the cumulative ranking curves for all interventions. Supplementary Fig 2. Comparison-adjusted funnel plot with pseudo 95% confidence limits. [file 12890_2024_3429_MOESM1_ESM.docx]

**Supplementary Text 1: Search strategies**

**Supplementary Text 2: Criteria for Study Inclusion and Exclusion**

**Supplementary Text 3: Definitions and criteria for primary and secondary outcomes.**

**Supplementary Text 4 and Supplementary Table 1: Risk of Bias Assessment**

**Supplementary Text 5 and Supplementary Table 2: Quality of Evidence Assessment**

**Supplementary Table 3: Sensitivity Analysis Results of SUCRA for Probability Ranking Plot and Odds Ratios for Pairwise Comparisons.**

**Supplementary Table 4: Results of Loop-specific approach.**

**Supplementary Table 5: Results of Side-splitting approach.**

**Supplementary Fig 1: Plots of the surface under the cumulative ranking curves for all interventions.**

**Supplementary Fig 2: Comparison-adjusted funnel plot with pseudo 95% confidence limits.**

**Supplementary Text 1: Search strategies**

MEDLINE:

(infant* OR infancy OR newborn* OR "new born*" OR "newly born" OR neonat* OR baby* OR babies OR premature* OR prematurity OR preterm* OR "pre term" OR premies OR "low birth weight" OR "low birthweight" OR NICU) AND (“less invasive surfactant administration” OR “minimally invasive surfactant treatment” OR “minimally invasive surfactant administration” OR “less invasive” OR “minimally invasive” OR “non-invasive” OR noninvasive OR “LISA” OR “MIST” OR “intubation-surfactant-extubation” OR “intubation, surfactant, extubation” OR “INSURE” OR nebuli* OR aerosol* OR atomiz* OR “surfactant administration” OR LMA OR “laryngeal mask airway” OR laryngeal OR catheter OR “feeding tube” OR angiocath OR magill) AND (surfactant* OR “surface active” OR “pulmonary surfactant*” OR “lung Surfactant*” OR “alveolar surfactant*”) AND (“randomized controlled trial” OR “controlled clinical trial” OR “random allocation” OR “double blind*” OR “single-blind*” OR “blind*” OR placebo OR trial* OR RCT OR random*)

Web of Science：

TS=(infant* OR infancy OR newborn* OR "new born*" OR “newly born” OR neonat* OR baby* OR babies OR premature* OR prematurity OR preterm* OR "pre term" OR premies or "low birth weight" OR "low birthweight" OR NICU) AND TS=(“less invasive surfactant administration” OR “minimally invasive surfactant treatment” OR “minimally invasive surfactant administration” OR “less invasive” OR “minimally invasive” OR “non invasive” OR noninvasive OR “LISA” OR “MIST” OR “intubation-surfactant-extubation” OR “intubation, surfactant, extubation” OR “INSURE” OR nebuli* OR aerosol* OR atomiz* OR “surfactant administration” OR “LMA” OR “laryngeal mask airway” OR “laryngeal” OR catheter OR “feeding tube” OR angiocath OR magill) AND TS=(surfactant* OR “surface active” OR “pulmonary surfactant*” OR “lung Surfactant*” OR “alveolar surfactant*”) AND TS=(“randomized controlled trial” OR “controlled clinical trial” OR “random allocation” OR “double blind*” OR “single blind*” OR “blind*” OR placebo OR trial* OR RCT OR random*)

Embase:

#1 'lung surfactant'/exp OR 'lung surfactant'

#2 surfactant* OR ‘surface active’ OR ‘pulmonary surfactant*’ OR ‘lung Surfactant*’ OR ‘alveolar surfactant*’

#3 #1 OR #2

#4 ‘less invasive surfactant administration’ OR 'minimally invasive surfactant treatment' OR 'minimally invasive surfactant administration' OR 'less invasive' OR 'minimally invasive' OR 'non-invasive' OR ‘noninvasive’ OR 'LISA' OR 'MIST' OR 'intubation-surfactant-extubation' OR 'intubation, surfactant, extubation' OR 'INSURE' OR nebuli* OR aerosol* OR atomiz* OR 'surfactant administration' OR 'LMA’ OR ‘laryngeal mask airway’ OR ‘laryngeal’ OR ‘catheter’ OR ‘feeding tube’ OR ‘angiocath’ OR ‘magill’

#5 infant* OR ‘infancy’ OR newborn* OR ‘new born*’ OR ‘newly born’ OR neonat* OR baby* OR ‘babies’ OR premature* OR ‘prematurity’ OR preterm* OR ‘pre-term’ OR ‘premies’ or ‘low birth weight’ OR ‘low birthweight’ OR ‘NICU’

#6 ‘randomized controlled trial’ OR ‘controlled clinical trial’ OR ‘random allocation’ OR ‘double blind’ OR ‘single blind’ OR blind* OR ‘placebo’ OR trial* OR ‘RCT’ OR random*

#7 #3 AND #4 AND #5 AND #6

Cochrane:

#1 MeSH descriptor: [Pulmonary Surfactants] explode all trees

#2 surfactant OR “surface active” OR pulmonary NEXT surfactant* OR lung NEXT surfactant* OR alveolar NEXT surfactant*

#3 #1 OR #2

#4 “less invasive surfactant administration” OR “minimally invasive surfactant treatment” OR “minimally invasive surfactant administration” OR “less invasive” OR “minimally invasive” OR “non invasive” OR noninvasive OR “LISA” OR “MIST” OR “intubation-surfactant-extubation” OR “intubation, surfactant, extubation” OR “INSURE” OR nebuli* OR aerosol* OR atomiz* OR “surfactant administration”

#5 infant* OR newborn OR new NEXT born* OR newly NEXT born* OR neonat* OR baby* OR babies OR premature* OR prematurity OR preterm OR “pre term” OR premies or “low birth weight” OR “low birthweight” OR NICU

#6 “randomized controlled trial” OR “controlled clinical trial” OR “random allocation” OR “double blind” OR “single blind” OR blind* OR placebo OR random* OR trial* OR RCT

#7 #3 AND #4 AND #5 AND #6

**Supplementary Text 2: Criteria for Study Inclusion and Exclusion**

The included infants were all clinically diagnosed or diagnosed on imaging with RDS within 24 hours of birth and did not receive intubation. We had no restriction on the gestational age of the included infants.

Specific definitions of the five ventilation strategies:

1. InSurE (Intubate, Surfactant, Extubate): intubation and surfactant administration followed by immediate extubation.

2. LISA (less invasive surfactant administration): a thin-diameter catheter is delivered into the infant's trachea with or without Magil forceps for surfactant delivery. All methods of PS administration via catheter have been combined into one single node, including the Cologne Method and the Hobart Method, and extensions based on these two methods.

3. LMA (laryngeal mask airway): delivering PS through a laryngeal mask airway, bypassing the need for laryngoscopy.

4. SN (surfactant nebulization): administration in the form of aerosolization, spreading evenly throughout the lungs by voluntary breathing.

5. UC (usual care): maintenance of simple nasal continuous positive airway pressure (CPAP) or nasal intermittent positive pressure ventilation (NIPPV) after birth, and selective intubation with administration of surfactant when non-invasive respiratory support fail.

**Supplementary Text 3: Definitions and criteria for primary and secondary outcomes.**

1. Intubation rate: Intubation rate, within the context of neonatal care during the initial seven days of life, refers to the frequency or proportion of newborns necessitating endotracheal intubation within this specified temporal framework. Criteria for intubation rate assessment could include predetermined clinical indicators such as respiratory distress syndrome severity, apnea with setting criteria of PIP, PEEP and FiO2, persistent hypoxemia with criteria of arterial blood gases (ABGs) (partial pressure of oxygen (PaO2), arterial oxygen saturation (SaO2), and the ratio of arterial oxygen tension to fractional inspired oxygen (PaO2/FiO2)), or escalating respiratory support requirements. These criteria served as evaluative benchmarks for healthcare professionals to ascertain the appropriateness of endotracheal intubation as a therapeutic intervention in neonates during the initial week of life.
2. Bronchopulmonary dysplasia (BPD): BPD is a chronic lung disease that primarily affects premature infants who have received mechanical ventilation and oxygen therapy. It is important to note that there are variations in the criteria used to diagnose and classify BPD, which may be influenced by evolving medical guidelines and research. Researchers typically use a combination of clinical, radiological and therapeutic parameters to determine the presence and severity of BPD in preterm infants. For this reason, we do not use uniform inclusion criteria to define BPD. Mild, moderate and severe BPD were all included.
3. Motaolity: Percentage of deaths occurring during hospitalisation
4. Severe intraventricular hemorrhage (IVH): IVH refers to a significant bleeding into the ventricles of the brain, often occurring in preterm infants. Children manifesting exceeding grade 2 were designated as a secondary outcome.
5. Retinopathy of prematurity (ROP): Retinopathy of Prematurity (ROP) is a proliferative retinal disorder primarily affecting premature infants, and laser therapy is a common intervention for its management. ROP requiring laser treatment was considered a secondary outcome.
6. Neonatal necrotizing enterocolitis (NEC): Necrotizing Enterocolitis (NEC) is a serious gastrointestinal disorder primarily affecting premature infants. NEC requiring surgical treatment was considered a secondary outcome.
7. Patent ductus arteriosus (PDA): Patent Ductus Arteriosus (PDA) is a congenital cardiovascular condition characterized by the persistence of a fetal blood vessel, the ductus arteriosus, connecting the pulmonary artery to the descending aorta after birth. PDA requiring surgical ligation is considered a secondary outcome.
8. Air leak: Including Pulmonary Interstitial Emphysema (PIE), Pneumothorax, Pneumomediastinum, Subcutaneous Emphysema, Bronchopleural Fistula and Alveolar Rupture.
9. Secondary dose administration: If the infant once again met the administration threshold following a fixed period of initial surfactant therapy, a second dose of surfactant was given by the same technique. The outcome criterion was met if the patient received two or more PS administrations.
10. Adverse events during surfactant administration: such as bradycardia, desaturation, regurgitation, cough, airway obstruction, asphyxia，hemodynamic instability，airway trauma.
11. Duration of mechanical ventilation.
12. Duration of oxygen support.
13. Length of hospital stay: The total duration a infant spends within the hospital environment, encompassing both general hospital care and Neonatal Intensive Care Unit (NICU) admission.

**Supplementary Text 4: Risk of Bias Assessment**

Utilize the Cochrane ROB tool to conduct a thorough evaluation of the risk of bias for each included RCT. The tool provided a structured framework for assessing the risk of bias in RCTs. The Cochrane ROB tool comprises the following domains:

1. Random Sequence Generation: Evaluate the adequacy of the randomization process;

2) Allocation Concealment: Assess whether sufficient measures were taken to prevent knowledge of the next allocation;

3) Blinding of Participants and Personnel: Evaluate whether appropriate blinding procedures were implemented;

4) Blinding of Outcome Assessment: Assess whether those assessing the outcomes were adequately blinded;

5) Incomplete Outcome Data:** Evaluate if there is a risk of selective outcome reporting;

6) Other Bias: Consider any other potential sources of bias.

Based on the above criteria for bias evaluation, we categorized the risk of bias as "low risk", "high risk" or "unclear risk". Integrate the risk of bias assessments for all included studies to determine the overall confidence level.

The results of the risk assessment are shown in the table below (eTable 1).

**Supplementary Table 1: Risk of bias of included trials**

|  | **Sequence generation** | **Allocation concealment** | **Blinding of participants and personnel** | **Follow-up Bias** | **Intubation** | **BPD** | **Death** | **IVH** | **PDA** | **NEC** | **ROP** | **Air leak** | **Adverse events** | **Secondary dose administration** | **Duration of mechanical ventilation** | **Duration of oxygen support** | **Length of hospital stay** |
| --- | --- | --- | --- | --- | --- | --- | --- | --- | --- | --- | --- | --- | --- | --- | --- | --- | --- |
| Mishra, A.,2023 | L | L | H^c^ | L | L | L | L | L | L | L | L | L | L | U | - | - | - |
| Kaleem, A.,2023 | L | U | U | L | U | - | L | L | - | - | - | L | - | L | U | - | U |
| Sabzehei, M. K.,2020 | L | L | L | L | L | U | - | L | L | L | L | L | L | U | U | - | U |
| A-nd, R,2020 | L | L | U | L | L | L | L | L | L | L | H^e^ | L | - | L | - | U | - |
| Pareek, P.,2021 | L | U | U | L | L | L | L | L | L | L | L | - | L | L | U | - | - |
| Akcay, N,2021 | U | U | U | H | U | U | U | U | U | H^e^ | - | U | - | U | - | - | - |
| Yang, G.,2020 | U | U | U | L | U | U | L | L | - | L | L | L | L | L | U | U | - |
| Han, T.,2020 | L | L | H^c^ | L | L | L | - | L | L | L | L |  | - | L | - | U | U |
| Gupta, B. K.,2020 | L | L | H^c^ | L | L | L | L | L | L | - | - | - | - | L | - | - | U |
| Je-, Soumya R.,2019 | L | L | H^c^ | L | L | L | L | L | L | U | - | L | L | L | - | - | - |
| Boskabadi, H.,2019 | L | U | H^c^ | U | U | U | - | L | - | - | - | - | - | - | - | - | U |
| Choupani, R.,2018 | L | U | L | U | L | U | L | L | - | - | - | - | L | L | - | - | - |
| Olivier, F.,2020 | L | L | H^c^ | U | L | - | - | - | - | - | - | L | L | U | U | - | - |
| Mosayebi, Z.,2017 | L | U | H^c^ | L | L | U | L | L | L | L | L | L | L | L | - | - | - |
| Mohammadizadeh M,2015 | L | L | H^c^ | L | L | L | L | L | - | - | - | - | L | L | - | U | - |
| Kribs, Angela,2015 | L | L | H^c^ | U | L | U | L | L | L | L | L | L | L | - | L | - | - |
| -yeri, F. S,2014 | U | U | L | U | L | U | L | L | L | - | - | L | - | - | - | - | - |
| Kanmaz, H. G.,2013 | L | L | H^c^ | U | L | L | L | L | L | L | L | L | L | U | U | U | - |
| Halim, A.,2019 | L | L | H^c^ | U | U | - | L | - | L | - | - | L | - | - | U | U | U |
| Goepel, Wolfgang,2011 | L | L | H^c^ | L | L | L | L | L | L | L | L | L | - | L | L | - | - |
| Kayvan Mirnia,2013 | U | U | U | U | U | U | L | L | L | L | L | L | L | H^f^ | U | U | U |
| Yingying Bao,2015 | U | U | U | U | L | U | - | L | L | L | L | L | L | U | U | U | U |
| Gaertner VD,2023 | L | L | H^c^ | U | L | L | L | L | L | L | L | L | L | - | - | - | - |
| Sadeghnia, A. R.,2020 | H^a^ | H^b^ | H^c^ | U | L | U | L | L | - | - | - | L | - | L | - | - | - |
| Cummings JJ,2020 | L | L | H^c^ | U | H^d^ | L | L | - | - | - | - | - | - | L | - | - | - |
| Kaluarachchi, D. C.,2023 | L | L | H^c^ | U | H^d^ | L | L | - | - | - | - | L | - | - | - | - | - |
| Minocchieri S,2019 | L | L | L | L | L | U | L | L | - | L | - | L | L | - | L | - | - |
| Dani C,2020 | L | U | H^c^ | L | U | L | L | L | L | L | L | L | L | L | U | U | - |
| Berggren E,2020 | U | L | H^c^ | U | U | L | - | - | L | - | - | L | - | - | - | - | - |
| NCT02074059, | U | U | H^c^ | U | - | - | L | - | - | L | - | L | L | - | - | - | - |
| Gallup, J. A.,2023 | L | L | H^c^ | L | L | L | L | - | - | L | - | L | L | L | - | - | U |
| Amini, E.,2019 | L | L | H^c^ | L | L | L | - | L | - | - | - | L | - | L | - | U | U |
| Roberts, K. D.,2017 | L | L | U | L | L | U | L | L | - | - | - | L | L | U | U | U | - |
| Gharehbaghi, M.,2018 | L | L | U | U | L | L | L | L | L | - | L | L | L | U | - | - | - |
| Pinheiro, J. M. B.,2015 | L | L | U | U | L | L | L | - | - | - | - | L | L | U | - | - | U |
| NCT02164734 | U | U | H^c^ | U | U | U | - | - | - | - | - | L | L | - | - | - | - |
| Attridge, J. T.,2013 | L | U | H^c^ | U | U | U | - | - | - | - | - | L | - | - | - | - | - |

Abbreviations: L, low risk; U, unclear risk; H, high risk.

a: Grouping based on odd or even number of files. For randomization, the patients with even file numbers were assigned to the control group, and those with an odd file number were assigned to the intervention group.

b: No allocation concealment.

C: No blinding of experiment implementers or operators.

D: Intubation criteria were based on clinical decisions by the implementer, who was not blinded.

E: The article did not report the number of infants for this outcome, although it was specified in the protocol, indicating a potential reporting bias.

F: The absence of criteria for secondary PS administration, coupled with non-blinding of the implementers, presumed to be high risk.

**Supplementary Text 5: Quality of Evidence Assessment**

We GRADEd each network estimate according to the five dimensions including Study limitation, Indirectness, Inconsistency, imprecision, and Publication bias. The criteria for downgrading are as follows.

**Study limitation:** Evidence with a low ROB contribution of less than 30 percent and moderate ROB contribution of more than 70 percent was downgraded by one quality level of evidence in terms of Study limitation.

**Imprecision:** A downgrade was also given if the point estimate of the effect value was greater than 1 but the lower bound was less than 0.8 or if the point estimate was less than 1 but the upper bound was greater than 1.25.

**Indirectness:** Systematically assess and compare the distribution of significant effect modifiers across comparisons, such as age, gender, etc., in a subjective way to determine their similarity across comparisons. Downgrade the quality of evidence if such similarity cannot be ensured. In addition, consider downgrading the quality of evidence if there are notable differences in subgroup analyses.

**Inconsistency:** Both heterogeneity and inconsistency are subject to potential downgrading, and this aspect can be downgraded by two levels. The determination of sufficient heterogeneity between each pair of head-to-head comparisons is assessed using prediction interval plots. The assessment of heterogeneity in the probability ranking plot uses the global τ-square. In line with Turner's findings, if the τ-square is less than 0.02, heterogeneity exists in the global network; conversely, if the τ-square is greater than 0.02, the overall evidence network is considered more homogeneous and no downgrading occurs due to heterogeneity. The assessment of inconsistency for each comparison is based on the results of both the ring inconsistency method and the node splitting method. The calculation of the p-value for global inconsistency is performed by frequency-based analysis. If the p-value is greater than 0.05, the global network is considered to be free of inconsistency; conversely, if the p-value is less than 0.05, inconsistency is identified and the quality of the probability ranking is downgraded.

**Publication bias:** The search strategy used for the INSURE and LISA comparisons was comprehensive and included both published and unpublished studies. Given the comprehensive nature of the search and the assessment that the likelihood of publication bias was low, no downgrading was applied. However, downgrading was applied to six comparisons with fewer than ten studies, excluding the INSURE and LISA comparisons.

**Supplementary Table 2: The results of the quality of evidence assessment.**

|  | **Study limitation** | **Indirectness** | **Inconsistency** | | **Imprecision** | **Publication bias** | **GRADE** |
| --- | --- | --- | --- | --- | --- | --- | --- |
|  |  |  | Heterogeneity | Inconsistency |  |  |  |
| **Intubation** |  |  |  |  |  |  |  |
| INSUREvsLISA | -1 | 0 | -1 | -1 | 0 | 0 | VERY LOW |
| INSUERvsLMA | -1 | 0 | 0 | 0 | 0 | -1 | LOW |
| INSUREvsSN | -1 | 0 | 0 | -1 | 0 | -1 | VERY LOW |
| INSUREvsUC | -1 | 0 | -1 | 0 | 0 | -1 | VERY LOW |
| LISAvsLMA | -1 | 0 | 0 | 0 | -1 | -1 | VERY LOW |
| LISAvsSN | -1 | 0 | -1 | 0 | 0 | -1 | VERY LOW |
| LISAvsUC | -1 | 0 | 0 | -1 | 0 | -1 | VERY LOW |
| LMAvsSN | -1 | 0 | -1 | 0 | 0 | -1 | VERY LOW |
| LMAvsUC | -1 | 0 | 0 | 0 | 0 | -1 | LOW |
| SNvsUC | -1 | 0 | 0 | -1 | 0 | -1 | VERY LOW |
| Rank | -1 | 0 | 0 | 0 | 0 | 0 | MODERATE |
| **BPD** |  |  |  |  |  |  |  |
| INSUREvsLISA | -1 | 0 | 0 | 0 | 0 | 0 | MODERATE |
| INSUERvsLMA | -1 | 0 | 0 | 0 | 0 | -1 | LOW |
| INSUREvsSN | -1 | 0 | 0 | 0 | -1 | -1 | VERY LOW |
| INSUREvsUC | -1 | 0 | 0 | 0 | -1 | -1 | VERY LOW |
| LISAvsLMA | -1 | 0 | 0 | 0 | 0 | -1 | LOW |
| LISAvsSN | -1 | 0 | 0 | 0 | 0 | -1 | LOW |
| LISAvsUC | -1 | 0 | 0 | 0 | 0 | -1 | LOW |
| LMAvsSN | -1 | 0 | 0 | 0 | -1 | -1 | VERY LOW |
| LMAvsUC | -1 | 0 | 0 | 0 | 0 | -1 | LOW |
| SNvsUC | -1 | 0 | 0 | 0 | -1 | -1 | VERY LOW |
| Rank | -1 | 0 | 0 | 0 | 0 | -1 | LOW |
| **Death** |  |  |  |  |  |  |  |
| INSUREvsLISA | 0 | 0 | 0 | 0 | 0 | 0 | HIGH |
| INSUERvsLMA | 0 | 0 | 0 | 0 | -1 | -1 | LOW |
| INSUREvsSN | 0 | 0 | 0 | 0 | -1 | -1 | LOW |
| INSUREvsUC | -1 | 0 | 0 | 0 | -1 | -1 | VERY LOW |
| LISAvsLMA | -1 | 0 | 0 | 0 | -1 | -1 | VERY LOW |
| LISAvsSN | 0 | 0 | 0 | 0 | -1 | -1 | LOW |
| LISAvsUC | 0 | 0 | 0 | 0 | -1 | -1 | LOW |
| LMAvsSN | 0 | 0 | 0 | 0 | -1 | -1 | LOW |
| LMAvsUC | 0 | 0 | 0 | 0 | -1 | -1 | LOW |
| SNvsUC | 0 | 0 | 0 | 0 | -1 | -1 | LOW |
| Rank | 0 | 0 | 0 | 0 | -1 | 0 | MODERATE |
| **Adverse events during surfactant administration** | | | | | | | |
| INSUREvsLISA | -1 | 0 | 0 | 0 | -1 | 0 | LOW |
| INSUERvsLMA | 0 | 0 | 0 | 0 | -1 | -1 | LOW |
| INSUREvsSN | 0 | 0 | 0 | 0 | -1 | -1 | LOW |
| INSUREvsUC | -1 | 0 | 0 | 0 | -1 | -1 | VERY LOW |
| LISAvsLMA | -1 | 0 | 0 | 0 | -1 | -1 | VERY LOW |
| LISAvsSN | 0 | 0 | 0 | 0 | -1 | -1 | LOW |
| LISAvsUC | 0 | 0 | 0 | 0 | -1 | -1 | LOW |
| LMAvsSN | 0 | 0 | 0 | 0 | -1 | -1 | LOW |
| LMAvsUC | -1 | 0 | 0 | 0 | -1 | -1 | VERY LOW |
| SNvsUC | 0 | 0 | 0 | 0 | -1 | -1 | LOW |
| Rank | 0 | 0 | -1 | 0 | -1 | 0 | LOW |
| **Secondary dose administration** | | | | | | | |
| INSUREvsLISA | -1 | 0 | 0 | 0 | 0 | 0 | MODERATE |
| INSUERvsLMA | -1 | 0 | 0 | 0 | -1 | -1 | VERY LOW |
| INSUREvsSN | 0 | 0 | 0 | -1 | -1 | -1 | VERY LOW |
| INSUREvsUC | -1 | 0 | 0 | 0 | 0 | -1 | LOW |
| LISAvsLMA | -1 | 0 | 0 | 0 | -1 | -1 | VERY LOW |
| LISAvsSN | -1 | 0 | 0 | 0 | -1 | -1 | VERY LOW |
| LISAvsUC | -1 | 0 | 0 | 0 | 0 | -1 | LOW |
| LMAvsSN | -1 | 0 | 0 | 0 | -1 | -1 | VERY LOW |
| LMAvsUC | -1 | 0 | 0 | 0 | 0 | -1 | LOW |
| SNvsUC | -1 | 0 | 0 | -1 | 0 | -1 | VERY LOW |
| Rank | -1 | 0 | 0 | -1 | 0 | 0 | LOW |
| **IVH** |  |  |  |  |  |  |  |
| INSUREvsLISA | -1 | 0 | 0 | 0 | -1 | 0 | LOW |
| INSUERvsLMA | 0 | 0 | 0 | 0 | -1 | -1 | LOW |
| INSUREvsSN | 0 | 0 | 0 | -1 | -1 | -1 | VERY LOW |
| INSUREvsUC | -1 | 0 | 0 | 0 | -1 | -1 | VERY LOW |
| LISAvsLMA | -1 | 0 | 0 | 0 | -1 | -1 | VERY LOW |
| LISAvsSN | -1 | 0 | 0 | 0 | -1 | -1 | VERY LOW |
| LISAvsUC | 0 | 0 | 0 | 0 | -1 | -1 | LOW |
| LMAvsSN | 0 | 0 | 0 | 0 | -1 | -1 | LOW |
| LMAvsUC | 0 | 0 | 0 | 0 | -1 | -1 | LOW |
| SNvsUC | 0 | 0 | 0 | -1 | -1 | -1 | VERY LOW |
| Rank | 0 | 0 | -1 | 0 | 0 | -1 | LOW |
| **Air leak** |  |  |  |  |  |  |  |
| INSUREvsLISA | -1 | 0 | 0 | 0 | -1 | 0 | LOW |
| INSUERvsLMA | 0 | 0 | 0 | 0 | -1 | -1 | LOW |
| INSUREvsSN | 0 | 0 | 0 | 0 | -1 | -1 | LOW |
| INSUREvsUC | 0 | 0 | 0 | 0 | 0 | -1 | MODERATE |
| LISAvsLMA | -1 | 0 | 0 | 0 | -1 | -1 | VERY LOW |
| LISAvsSN | 0 | 0 | 0 | 0 | 0 | -1 | MODERATE |
| LISAvsUC | 0 | 0 | 0 | 0 | 0 | -1 | MODERATE |
| LMAvsSN | 0 | 0 | 0 | 0 | -1 | -1 | LOW |
| LMAvsUC | 0 | 0 | 0 | 0 | -1 | -1 | LOW |
| SNvsUC | 0 | 0 | 0 | 0 | -1 | -1 | LOW |
| Rank | 0 | 0 | 0 | 0 | 0 | 0 | HIGH |
| **NEC** |  |  |  |  |  |  |  |
| INSUREvsLISA | 0 | 0 | 0 | 0 | -1 | 0 | MODERATE |
| INSUERvsLMA | 0 | 0 | 0 | 0 | -1 | -1 | LOW |
| INSUREvsSN | 0 | 0 | 0 | 0 | -1 | -1 | LOW |
| INSUREvsUC | 0 | 0 | 0 | 0 | -1 | -1 | LOW |
| LISAvsLMA | 0 | 0 | 0 | 0 | -1 | -1 | LOW |
| LISAvsSN | 0 | 0 | 0 | 0 | -1 | -1 | LOW |
| LISAvsUC | 0 | 0 | 0 | 0 | -1 | -1 | LOW |
| LMAvsSN | 0 | 0 | 0 | 0 | -1 | -1 | LOW |
| LMAvsUC | 0 | 0 | 0 | 0 | -1 | -1 | LOW |
| SNvsUC | 0 | 0 | 0 | 0 | -1 | -1 | LOW |
| Rank | 0 | 0 | 0 | 0 | -1 | 0 | MODERATE |
| **PDA** |  |  |  |  |  |  |  |
| INSUREvsLISA | -1 | 0 | 0 | 0 | 0 | 0 | MODERATE |
| INSUERvsLMA | 0 | 0 | 0 | -1 | -1 | -1 | VERY LOW |
| INSUREvsSN | 0 | 0 | 0 | 0 | 0 | -1 | MODERATE |
| INSUREvsUC | -1 | 0 | 0 | -1 | 0 | -1 | VERY LOW |
| LISAvsLMA | -1 | 0 | 0 | 0 | -1 | -1 | VERY LOW |
| LISAvsSN | 0 | 0 | -1 | 0 | 0 | -1 | MODERATE |
| LISAvsUC | 0 | 0 | 0 | -1 | 0 | -1 | LOW |
| LMAvsSN | 0 | 0 | 0 | 0 | -1 | -1 | LOW |
| LMAvsUC | 0 | 0 | 0 | 0 | -1 | -1 | LOW |
| SNvsUC | 0 | 0 | 0 | 0 | -1 | -1 | LOW |
| Rank | 0 | 0 | -1 | 0 | 0 | 0 | MODERATE |
| **ROP** |  |  |  |  |  |  |  |
| INSUREvsLISA | 0 | 0 | 0 | 0 | -1 | -1 | LOW |
| INSUERvsLMA | 0 | 0 | 0 | 0 | -1 | -1 | LOW |
| INSUREvsSN | 0 | 0 | 0 | 0 | -1 | -1 | LOW |
| INSUREvsUC | 0 | 0 | 0 | 0 | -1 | -1 | LOW |
| LISAvsLMA | 0 | 0 | 0 | 0 | -1 | -1 | LOW |
| LISAvsSN | 0 | 0 | 0 | 0 | -1 | -1 | LOW |
| LISAvsUC | 0 | 0 | 0 | 0 | -1 | -1 | LOW |
| LMAvsSN | 0 | 0 | 0 | 0 | -1 | -1 | LOW |
| LMAvsUC | 0 | 0 | 0 | 0 | -1 | -1 | LOW |
| SNvsUC | 0 | 0 | 0 | 0 | -1 | -1 | LOW |
| Rank | 0 | 0 | 0 | 0 | -1 | -1 | LOW |
| **Duration of mechanical ventilation** | | | | | | | |
| INSUREvsLISA | -1 | 0 | 0 | 0 | -1 | -1 | VERY LOW |
| INSUERvsLMA | -1 | 0 | 0 | 0 | -1 | -1 | VERY LOW |
| INSUREvsSN | -1 | 0 | 0 | 0 | -1 | -1 | VERY LOW |
| INSUREvsUC | -1 | 0 | 0 | 0 | -1 | -1 | VERY LOW |
| LISAvsLMA | -1 | 0 | 0 | 0 | -1 | -1 | VERY LOW |
| LISAvsSN | -1 | 0 | 0 | 0 | -1 | -1 | VERY LOW |
| LISAvsUC | -1 | 0 | 0 | 0 | -1 | -1 | VERY LOW |
| LMAvsSN | -1 | 0 | 0 | 0 | -1 | -1 | VERY LOW |
| LMAvsUC | -1 | 0 | 0 | 0 | -1 | -1 | VERY LOW |
| SNvsUC | -1 | 0 | 0 | 0 | -1 | -1 | VERY LOW |

Abbreviations: InSurE, Intubate, Surfactant, Extubate; LISA, less invasive surfactant administration; LMA, laryngeal mask airway; SN, surfactant nebulization; UC,usual care; BPD, Bronchopulmonary dysplasia; IVH, Severe intraventricular hemorrhage; ROP, Retinopathy of prematurity; NEC, Neonatal necrotizing enterocolitis; PDA, Patent ductus arteriosus.

**Supplementary Table 3: Sensitivity Analysis Results of SUCRA for Probability Ranking Plot and Odds Ratios for Pairwise Comparisons**

**A: Analysis of removal of high-risk research (including company-sponsored research).**

| **nCPAP** | **BPD** | | | | | **SUCRA** |
| --- | --- | --- | --- | --- | --- | --- |
| **Intubation** | **InSurE** | 1.61 (1.21,2.16) | 0.62 (0.29,1.30) | 0.59 (0.08,4.10) | 1.06 (0.61,1.86) | 50.4 |
|  | 1.85 (1.37,2.49) | **LISA** | 0.38 (0.18,0.83) | 0.36 (0.05,2.50) | 0.66 (0.40,1.08) | 95.1 |
|  | 1.49 (0.77,2.89) | 0.81 (0.40,1.62) | **LMA** | 0.95 (0.12,7.24) | 1.72 (0.76,3.88) | 18.2 |
|  | 0.35 (0.11,1.13) | 0.19 (0.06,0.61) | 0.23 (0.07,0.80) | **SN** | 1.81 (0.28,11.60) | 29.4 |
|  | 0.26 (0.13,0.50) | 0.14 (0.07,0.27) | 0.17 (0.08,0.36) | 0.73 (0.29,1.89) | **UC** | 56.9 |
| **SUCRA** | 52.0 | 93.0 | 78.7 | 20.0 | 6.4 |  |
| **nCPAP** | **IVH** | | | | | **SUCRA** |
| **Death** | **InSurE** | 1.04 (0.69,1.55) | 0.92 (0.20,4.19) | 2.92 (0.64,13.39) | 0.59 (0.28,1.27) | 49.2 |
|  | 1.30 (0.95,1.78) | **LISA** | 0.89 (0.19,4.21) | 2.82 (0.60,13.38) | 0.57 (0.29,1.14) | 53.7 |
|  | 1.58 (0.24,10.24) | 1.21 (0.18,8.00) | **LMA** | 3.18 (0.38,26.94) | 0.64 (0.12,3.34) | 44.4 |
|  | 1.18 (0.11,12.96) | 0.90 (0.08,9.84) | 0.74 (0.04,15.02) | **SN** | 0.20 (0.04,1.02) | 91.5 |
|  | 1.15 (0.57,2.34) | 0.89 (0.45,1.73) | 0.73 (0.10,5.09) | 0.98 (0.10,9.72) | **UC** | 11.5 |
| **SUCRA** | 28.2 | 63.3 | 61.1 | 49.9 | 47.5 |  |
| **nCPAP** | **Adverse events** | | | | | **SUCRA** |
| **Air leak** | **InSurE** | 1.14 (0.62,2.09) | 1.37 (0.47,4.03) | 1.37 (0.05,35.75) | 1.33 (0.40,4.41) | 34.1 |
|  | 1.31 (0.76,2.24) | **LISA** | 1.20 (0.36,3.97) | 1.20 (0.05,29.93) | 1.17 (0.40,3.40) | 47.8 |
|  | 0.77 (0.36,1.61) | 0.59 (0.25,1.37) | **LMA** | 1.00 (0.03,29.28) | 0.97 (0.22,4.29) | 59.6 |
|  | 0.51 (0.21,1.26) | 0.39 (0.16,0.94) | 0.66 (0.24,1.86) | **SN** | 0.97 (0.05,20.23) | 52.2 |
|  | 0.54 (0.27,1.09) | 0.42 (0.22,0.79) | 0.71 (0.31,1.63) | 1.07 (0.57,2.02) | **UC** | 56.3 |
| **SUCRA** | 70.6 | 92.7 | 47.9 | 18.1 | 20.7 |  |
| **nCPAP** |  | | | | |  |
| **Secondary dose** | **InSurE** |  |  |  |  |  |
|  | 0.95 (0.71,1.27) | **LISA** |  |  |  |  |
|  | 0.82 (0.40,1.67) | 0.87 (0.41,1.82) | **LMA** |  |  |  |
|  | 1.19 (0.38,3.72) | 1.25 (0.38,4.05) | 1.44 (0.37,5.53) | **SN** |  |  |
|  | 2.05 (0.84,5.00) | 2.16 (0.88,5.27) | 2.49 (1.15,5.41) | 1.73 (0.41,7.37) | **UC** |  |
| **SUCRA** | 43.9 | 35.3 | 24.6 | 54.5 | 91.7 |  |

**B: The results of re-analysis studies with FiO2 values between 0.3 and 0.4.**

| **nCPAP** | **BPD** | | | | | **SUCRA** |
| --- | --- | --- | --- | --- | --- | --- |
| **Intubation** | **InSurE** | 1.64 (1.19,2.25) | 0.20 (0.02,2.00) | 1.43 (0.35,5.84) | 1.15 (0.60,2.20) | 39.4 |
|  | 1.86 (1.41,2.45) | **LISA** | 0.12 (0.01,1.20) | 0.87 (0.21,3.63) | 0.70 (0.40,1.24) | 85.5 |
|  | 0.79 (0.23,2.78) | 0.43 (0.12,1.46) | **LMA** | 7.17 (0.50,103.50) | 5.78 (0.63,52.84) | 6.5 |
|  | 0.75 (0.23,2.47) | 0.40 (0.12,1.33) | 0.95 (0.20,4.49) | **SN** | 0.81 (0.18,3.58) | 66.1 |
|  | 0.27 (0.13,0.58) | 0.15 (0.07,0.30) | 0.34 (0.13,0.93) | 0.36 (0.11,1.19) | **UC** | 52.5 |
| **SUCRA** | 57.8 | 96.1 | 48.9 | 45.8 | 1.5 |  |
| **nCPAP** | **IVH** | | | | | **SUCRA** |
| **Death** | **InSurE** | 1.08 (0.71,1.64) | 0.66 (0.01,39.41) | 2.88 (0.45,18.26) | 0.70 (0.28,1.73) | 46.7 |
|  | 1.37 (0.97,1.94) | **LISA** | 0.62 (0.01,35.78) | 2.67 (0.40,17.80) | 0.65 (0.30,1.43) | 56.4 |
|  | 1.44 (0.03,80.27) | 1.05 (0.02,57.65) | **LMA** | 4.34 (0.05,384.82) | 1.06 (0.02,57.00) | 38.4 |
|  | 2.09 (0.18,24.61) | 1.52 (0.13,18.39) | 1.45 (0.01,163.08) | **SN** | 0.24 (0.03,1.91) | 84.2 |
|  | 1.52 (0.67,3.46) | 1.11 (0.53,2.34) | 1.06 (0.02,54.40) | 0.73 (0.05,9.82) | **UC** | 24.3 |
| **SUCRA** | 23.0 | 55.3 | 49.8 | 63.1 | 58.8 |  |
| **nCPAP** | **Adverse events** | | | | | **SUCRA** |
| **Air leak** | **InSurE** | 1.16 (0.58,2.31) | 4.33 (0.13,147.75) | - | 2.25 (0.42,12.18) | 23.5 |
|  | 1.40 (0.82,2.40) | **LISA** | 3.73 (0.12,119.10) | - | 1.94 (0.41,9.09) | 36.0 |
|  | 0.74 (0.14,3.89) | 0.53 (0.11,2.57) | **LMA** | - | - | 75.4 |
|  | 0.89 (0.20,3.94) | 0.63 (0.15,2.73) | 1.20 (0.17,8.34) | **SN** | 0.52 (0.02,11.55) | - |
|  | 0.62 (0.25,1.56) | 0.44 (0.20,0.96) | 0.83 (0.21,3.30) | 0.70 (0.18,2.75) | **UC** | 65.2 |
| **SUCRA** | 52.7 | 83.9 | 41.7 | 49.3 | 22.3 |  |

**C: The results of re-analysis studies with PEEP levels between 5 and 8 cmH2O.**

| **nCPAP** | **BPD** | | | | | **SUCRA** |
| --- | --- | --- | --- | --- | --- | --- |
| **Intubation** | **InSurE** | 1.99 (1.34,2.94) | 0.75 (0.13,4.24) | 1.77 (0.40,7.80) | 1.62 (0.67,3.91) | 25.3 |
|  | 1.97 (1.33,2.90) | **LISA** | 0.38 (0.07,2.13) | 0.89 (0.21,3.73) | 0.81 (0.37,1.80) | 78.5 |
|  | 0.91 (0.33,2.53) | 0.46 (0.16,1.31) | **LMA** | 2.37 (0.29,19.08) | 2.16 (0.39,11.99) | 22.7 |
|  | 0.19 (0.05,0.77) | 0.10 (0.03,0.38) | 0.21 (0.06,0.76) | **SN** | 0.91 (0.28,3.01) | 63.5 |
|  | 0.19 (0.06,0.58) | 0.10 (0.03,0.29) | 0.21 (0.08,0.56) | 1.00 (0.44,2.26) | **UC** | 60.0 |
| **SUCRA** | 63.8 | 98.0 | 62.5 | 13.2 | 12.5 |  |
| **nCPAP** | **IVH** | | | | | **SUCRA** |
| **Death** | **InSurE** | 1.17 (0.68,2.01) | 0.45 (0.01,28.51) | 0.26 (0.02,2.80) | 0.47 (0.15,1.50) | 67.9 |
|  | 1.64 (0.93,2.89) | **LISA** | 0.38 (0.01,23.48) | 0.22 (0.02,2.24) | 0.40 (0.15,1.12) | 81.2 |
|  | 2.41 (0.19,30.28) | 1.47 (0.12,18.75) | **LMA** | 0.58 (0.01,52.43) | 1.06 (0.02,57.46) | 43.9 |
|  | 3.99 (0.27,59.78) | 2.44 (0.17,34.49) | 1.65 (0.05,59.81) | **SN** | 1.82 (0.23,14.62) | 23.5 |
|  | 1.34 (0.48,3.76) | 0.82 (0.34,1.96) | 0.55 (0.04,7.26) | 0.34 (0.03,4.10) | **UC** | 33.6 |
| **SUCRA** | 18.8 | 57.3 | 60.7 | 74.8 | 38.3 |  |
| **nCPAP** | **Adverse events** | | | | | **SUCRA** |
| **Air leak** | **InSurE** | 0.92 (0.44,1.92) | 2.07 (0.36,11.80) | 2.74 (0.27,28.23) | 1.28 (0.28,5.92) | 34.3 |
|  | 1.41 (0.74,2.67) | **LISA** | 2.25 (0.37,13.83) | 2.98 (0.32,28.10) | 1.40 (0.34,5.67) | 26.7 |
|  | 0.84 (0.30,2.37) | 0.59 (0.20,1.77) | **LMA** | 1.33 (0.09,18.74) | 0.62 (0.09,4.49) | 67.0 |
|  | 1.21 (0.15,9.90) | 0.86 (0.11,6.64) | 1.44 (0.17,12.25) | **SN** | 0.47 (0.08,2.71) | 75.9 |
|  | 0.56 (0.20,1.52) | 0.40 (0.16,0.96) | 0.67 (0.23,1.96) | 0.46 (0.07,2.93) | **UC** | 46.1 |
| **SUCRA** | 51.9 | 80.2 | 42.6 | 60.9 | 14.3 |  |
| **nCPAP** |  | | | | |  |
| **Secondary dose** | **InSurE** |  |  |  |  |  |
|  | 0.95 (0.69,1.33) | **LISA** |  |  |  |  |
|  | 1.60 (0.59,4.36) | 1.68 (0.61,4.62) | **LMA** |  |  |  |
|  | 0.38 (0.02,8.08) | 0.40 (0.02,8.43) | 0.24 (0.01,4.74) | **SN** |  |  |
|  | 3.23 (1.17,8.93) | 3.38 (1.23,9.26) | 2.02 (0.90,4.51) | 8.53 (0.48,152.98) | **UC** |  |
| **SUCRA** | 38.4 | 31.7 | 63.4 | 20.0 | 96.5 |  |

**D: The results of re-analysis subgroup analysis by dosing regimen (100 mg/Kg and 200 mg/Kg).**

| **Intubation** | **200mg/Kg** | | | | | **SUCRA** |
| --- | --- | --- | --- | --- | --- | --- |
| **100mg/Kg** | **InSurE** | 1.50 (1.06,2.12) | 0.82 (0.24,2.81) | 0.75 (0.16,3.43) | 0.28 (0.06,1.22) | 55.3 |
|  | 2.03 (1.20,3.42) | **LISA** | 0.55 (0.15,1.96) | 0.50 (0.11,2.37) | 0.19 (0.04,0.85) | 90.4 |
|  | 2.78 (1.11,6.99) | 1.37 (0.48,3.89) | **LMA** | 0.91 (0.38,2.23) | 0.34 (0.15,0.76) | 53.5 |
|  | 1.30 (0.40,4.22) | 0.64 (0.19,2.13) | 0.47 (0.11,2.03) | **SN** | 0.37 (0.25,0.55) | 49.1 |
|  | 0.25 (0.11,0.54) | 0.12 (0.06,0.26) | 0.09 (0.03,0.28) | 0.19 (0.06,0.59) | **UC** | 1.7 |
| **SUCRA** | 33.6 | 76.0 | 88.9 | 51.4 | 0.0 |  |
| **BPD** | **200mg/Kg** | | | | | **SUCRA** |
| **100mg/Kg** | **InSurE** | 1.37 (0.83,2.25) | 0.46 (0.08,2.75) | 2.17 (0.13,37.79) | 2.68 (0.16,44.87) | 36.3 |
|  | 1.40 (0.96,2.04) | **LISA** | 0.34 (0.05,2.16) | 1.59 (0.09,28.91) | 1.96 (0.11,34.35) | 61.5 |
|  | 0.76 (0.32,1.78) | 0.54 (0.22,1.32) | **LMA** | 4.68 (0.50,43.69) | 5.78 (0.65,51.31) | 12.1 |
|  | 2.06 (0.50,8.48) | 1.47 (0.34,6.30) | 2.72 (0.52,14.14) | **SN** | 1.23 (0.77,1.97) | 60.5 |
|  | 0.91 (0.51,1.65) | 0.65 (0.40,1.06) | 1.21 (0.47,3.09) | 0.44 (0.10,2.00) | **UC** | 79.6 |
| **SUCRA** | 38.8 | 78.0 | 20.5 | 81.9 | 30.8 |  |
| **Death** | **200mg/Kg** | | | | | **SUCRA** |
| **100mg/Kg** | **InSurE** | - | - | - | - | - |
|  | 1.20 (0.81,1.77) | **LISA** | - | - | - | - |
|  | 1.76 (0.21,14.54) | 1.46 (0.17,12.55) | **LMA** | - | - | - |
|  | 2.09 (0.18,24.61) | 1.74 (0.14,21.13) | 1.19 (0.05,30.59) | **SN** | - | - |
|  | 1.07 (0.51,2.25) | 0.89 (0.45,1.75) | 0.61 (0.06,5.72) | 0.51 (0.04,6.74) | **UC** | - |
| **SUCRA** | 29.4 | 54.0 | 61.9 | 65.5 | 39.3 |  |
| **IVH** | **200mg/Kg** | | | | | **SUCRA** |
| **100mg/Kg** | **InSurE** | 1.23 (0.57,2.64) | 1.00 (0.19,5.40) | 1.12 (0.00,386.55) | 1.06 (0.01,76.90) | 44.0 |
|  | 0.97 (0.54,1.72) | **LISA** | 0.81 (0.13,5.18) | 0.91 (0.00,330.10) | 0.86 (0.01,66.85) | 58.3 |
|  | 1.00 (0.02,57.70) | 1.03 (0.02,62.11) | **LMA** | 1.12 (0.00,301.42) | 1.06 (0.02,54.40) | 46.8 |
|  | 2.08 (0.35,12.26) | 2.15 (0.34,13.50) | 2.08 (0.02,174.05) | **SN** | 0.95 (0.02,50.32) | 51.0 |
|  | 0.52 (0.21,1.32) | 0.54 (0.24,1.22) | 0.52 (0.01,33.46) | 0.25 (0.04,1.71) | **UC** | 49.9 |
| **SUCRA** | 54.3 | 52.0 | 50.0 | 78.6 | 15.1 |  |
| **Air leak** | **200mg/Kg** | | | | | **SUCRA** |
| **100mg/Kg** | **InSurE** | 1.15 (0.53,2.51) | 0.19 (0.01,4.06) | 0.14 (0.00,4.70) | 0.16 (0.01,4.55) | 73.6 |
|  | 1.39 (0.69,2.79) | **LISA** | 0.16 (0.01,3.87) | 0.12 (0.00,4.43) | 0.14 (0.00,4.30) | 81.6 |
|  | 0.90 (0.38,2.12) | 0.65 (0.22,1.87) | **LMA** | 0.75 (0.14,4.05) | 0.83 (0.21,3.30) | 37.2 |
|  | 2.29 (0.33,16.14) | 1.65 (0.22,12.23) | 2.55 (0.31,21.13) | **SN** | 1.11 (0.42,2.92) | 26.2 |
|  | 0.50 (0.22,1.14) | 0.36 (0.18,0.71) | 0.55 (0.18,1.69) | 0.22 (0.03,1.59) | **UC** | 31.0 |
| **SUCRA** | 48.4 | 72.9 | 41.6 | 80.5 | 6.6 |  |
| **Adverse events** | **200mg/Kg** | | | | | **SUCRA** |
| **100mg/Kg** | **InSurE** | 1.14 (0.63,2.08) | 1.38 (0.47,4.00) | 2.90 (0.37,22.85) | 1.33 (0.41,4.35) | 56.0 |
|  | 0.80 (0.45,1.43) | **LISA** | 1.20 (0.37,3.93) | 2.53 (0.35,18.58) | 1.17 (0.40,3.36) | 80.4 |
|  | 1.88 (0.86,4.09) | 2.35 (0.87,6.33) | **LMA** | 2.11 (0.23,19.69) | 0.97 (0.22,4.22) | 42.9 |
|  | 0.95 (0.02,55.95) | 1.19 (0.02,66.79) | 0.51 (0.01,32.29) | **SN** | 0.46 (0.09,2.49) | 38.5 |
|  | 0.95 (0.39,2.33) | 1.19 (0.63,2.27) | 0.51 (0.15,1.70) | 1.00 (0.02,53.17) | **UC** | 32.2 |
| **SUCRA** | 46.2 | 25.9 | 84.4 | 48.6 | 44.9 |  |
| **Secondary dose** | **200mg/Kg** | | | | | **SUCRA** |
| **100mg/Kg** | **InSurE** | 0.93 (0.65,1.33) | 1.01 (0.46,2.18) | 0.54 (0.17,1.75) | 3.24 (1.24,8.47) | 50.6 |
|  | 0.86 (0.57,1.32) | **LISA** | 1.08 (0.48,2.45) | 0.59 (0.17,2.02) | 3.49 (1.35,9.03) | 39.1 |
|  | 0.87 (0.38,1.98) | 1.00 (0.40,2.53) | **LMA** | 0.54 (0.14,2.12) | 3.22 (1.35,7.71) | 47.1 |
|  | 1.19 (0.36,3.90) | 1.37 (0.39,4.86) | 1.37 (0.32,5.84) | **SN** | 5.95 (1.41,25.11) | 14.1 |
|  | 1.66 (0.41,6.79) | 1.92 (0.50,7.36) | 1.92 (0.37,9.79) | 1.40 (0.22,8.87) | **UC** | 99.2 |
| **SUCRA** | 51.1 | 30.9 | 35.5 | 57.5 | 75.0 |  |

**E: The results of re-analysis subgroup analysis by gestational age (28-32 weeks and >32 weeks).**

| **Intubation** | **>32w** | | | | | **SUCRA** |
| --- | --- | --- | --- | --- | --- | --- |
| **28-32w** | **InSurE** | 1.34 (0.70,2.56) | 0.56 (0.20,1.56) | 0.47 (0.15,1.49) | 0.16 (0.05,0.48) | 74.0 |
|  | 1.90 (1.31,2.76) | **LISA** | 0.42 (0.14,1.23) | 0.35 (0.11,1.12) | 0.12 (0.04,0.36) | 92.9 |
|  | 3.29 (1.20,8.98) | 1.73 (0.59,5.04) | **LMA** | 0.83 (0.37,1.88) | 0.29 (0.14,0.59) | 46.2 |
|  | 0.68 (0.19,2.44) | 0.36 (0.10,1.35) | 0.21 (0.04,1.05) | **SN** | 0.35 (0.23,0.52) | 37.0 |
|  | 0.55 (0.16,1.93) | 0.29 (0.08,1.07) | 0.17 (0.03,0.83) | 0.80 (0.38,1.70) | **UC** | 0 |
| **SUCRA** | 39.2 | 76.5 | 94.8 | 27.1 | 12.4 |  |
| **BPD** | **>32w** | | | | | **SUCRA** |
| **28-32w** | **InSurE** | 1.52 (0.93,2.46) | 0.53 (0.10,2.68) | 2.45 (0.16,38.86) | 3.05 (0.20,46.37) | 44.8 |
|  | 1.52 (0.93,2.46) | **LISA** | 0.35 (0.06,1.90) | 1.62 (0.10,26.74) | 2.01 (0.13,31.93) | 77.4 |
|  | 0.53 (0.10,2.68) | 0.35 (0.06,1.90) | **LMA** | 4.65 (0.50,43.43) | 5.78 (0.65,51.31) | 15.6 |
|  | 0.95 (0.11,7.85) | 0.63 (0.07,5.47) | 1.79 (0.46,6.96) | **SN** | 1.24 (0.77,2.00) | 45.1 |
|  | 1.18 (0.15,9.25) | 0.78 (0.09,6.46) | 2.23 (0.63,7.94) | 1.24 (0.77,2.00) | **UC** | 67.1 |
| **SUCRA** | 35.2 | 74.2 | 31.8 | 58.3 | 50.4 |  |
| **Death** | **>32w** | | | | | **SUCRA** |
| **28-32w** | **InSurE** | 1.26 (0.35,4.53) | 1.00 (0.02,52.36) | 3.23 (0.01,2023.61) | 1.06 (0.00,281.94) | 43.8 |
|  | 1.36 (0.98,1.89) | **LISA** | 0.79 (0.01,50.79) | 2.57 (0.00,1819.79) | 0.84 (0.00,258.35) | 53.0 |
|  | 2.20 (0.18,26.79) | 1.62 (0.13,20.10) | **LMA** | 3.23 (0.02,519.22) | 1.06 (0.02,54.40) | 43.9 |
|  | 1.25 (0.22,7.07) | 0.92 (0.16,5.35) | 0.57 (0.03,11.90) | **SN** | 0.33 (0.01,8.08) | 65.9 |
|  | 0.51 (0.13,2.03) | 0.37 (0.09,1.55) | 0.23 (0.01,4.03) | 0.41 (0.08,1.96) | **UC** | 43.3 |
| **SUCRA** | 38.5 | 69.1 | 72.0 | 57.3 | 13.1 |  |
| **IVH** | **>32w** | | | | | **SUCRA** |
| **28-32w** | **InSurE** | 1.09 (0.27,4.40) | 1.00 (0.17,5.99) | - | 1.06 (0.01,91.01) | 48.5 |
|  | 1.21 (0.77,1.89) | **LISA** | - | 0.91 (0.09,8.82) | 0.97 (0.01,102.81) | 52.3 |
|  | 0.96 (0.22,4.13) | - | **LMA** | - | - | 48.7 |
|  | - | 0.80 (0.17,3.66) | - | **SN** | 1.06 (0.02,62.52) | - |
|  | 0.39 (0.09,1.65) | 0.33 (0.07,1.46) | - | 0.41 (0.09,1.96) | **UC** | 50.4 |
| **SUCRA** | 41.5 | 56.3 | - | 84.4 | 17.9 |  |
| **Air leak** | **>32w** | | | | | **SUCRA** |
| **28-32w** | **InSurE** | 1.45 (0.34,6.28) | 0.54 (0.08,3.73) | 0.34 (0.04,3.19) | 0.38 (0.05,2.81) | 68.0 |
|  | 1.29 (0.72,2.32) | **LISA** | 0.37 (0.05,2.75) | 0.23 (0.03,2.11) | 0.26 (0.04,1.85) | 83.6 |
|  | 0.81 (0.32,2.05) | 0.62 (0.21,1.87) | **LMA** | 0.62 (0.13,2.91) | 0.71 (0.23,2.21) | 46.6 |
|  | 0.63 (0.15,2.65) | 0.49 (0.10,2.30) | 0.78 (0.14,4.33) | **SN** | 1.13 (0.40,3.18) | 23.3 |
|  | 0.67 (0.18,2.52) | 0.51 (0.12,2.20) | 0.83 (0.16,4.19) | 1.05 (0.47,2.39) | **UC** | 28.5 |
| **SUCRA** | 58.6 | 81.3 | 43.1 | 31.7 | 35.3 |  |
| **Adverse events** | **>32w** | | | | | **SUCRA** |
| **28-32w** | **InSurE** | 1.27 (0.62,2.60) | 0.56 (0.13,2.40) | - | 0.56 (0.14,2.28) | 61.1 |
|  | 1.07 (0.45,2.53) | **LISA** | - | 0.44 (0.09,2.06) | 0.44 (0.12,1.59) | 83.2 |
|  | 2.09 (0.41,10.53) | 1.95 (0.31,12.15) | **LMA** | - | - | 28.7 |
|  | 4.30 (0.31,60.41) | 4.01 (0.33,48.59) | 2.06 (0.09,45.68) | **SN** | 1.00 (0.18,5.51) | - |
|  | 2.10 (0.34,13.07) | 1.96 (0.39,9.88) | 1.01 (0.09,11.56) | 0.49 (0.07,3.28) | **UC** | 26.9 |
| **SUCRA** | 24.7 | 29.2 | 60.3 | 79.1 | 56.7 |  |
| **Secondary dose** | **>32w** | | | | | **SUCRA** |
| **28-32w** | **InSurE** | 0.73 (0.42,1.27) | 0.48 (0.11,2.13) | 0.01 (0.00,0.13) | 1.31 (0.35,4.83) | 76.1 |
|  | 0.97 (0.70,1.34) | **LISA** | 0.66 (0.16,2.75) | 0.01 (0.00,0.17) | 1.80 (0.53,6.06) | 50.6 |
|  | 0.92 (0.40,2.07) | 0.94 (0.39,2.27) | **LMA** | 0.01 (0.00,0.22) | 2.72 (1.14,6.47) | 36.3 |
|  | 1.19 (0.38,3.72) | 1.22 (0.37,4.01) | 1.30 (0.32,5.28) | **SN** | 225.29 (13.86,3661.86) | 0.0 |
|  | 10.11 (0.45,225.51) | 10.42 (0.46,236.42) | 11.05 (0.45,273.82) | 8.53 (0.48,152.98) | **UC** | 86.9 |
| **SUCRA** | 39.8 | 35.5 | 32.8 | 48.8 | 93 |  |

**F: The results of re-analysis studies with CPAP as the respiratory support mode.**

| **nCPAP** | **BPD** | | | | | **SUCRA** |
| --- | --- | --- | --- | --- | --- | --- |
| **Intubation** | **InSurE** | 1.74 (1.28,2.37) | 0.65 (0.31,1.35) | 1.07 (0.57,2.03) | 1.23 (0.73,2.07) | 37.7 |
|  | 2.31 (1.56,3.40) | **LISA** | 0.37 (0.17,0.80) | 0.62 (0.34,1.12) | 0.71 (0.45,1.12) | 96.7 |
|  | 1.70 (0.82,3.49) | 0.74 (0.34,1.61) | **LMA** | 1.65 (0.68,4.01) | 1.90 (0.85,4.22) | 7.9 |
|  | 0.61 (0.26,1.44) | 0.26 (0.11,0.63) | 0.36 (0.13,0.96) | **SN** | 1.15 (0.76,1.75) | 44.3 |
|  | 0.36 (0.18,0.70) | 0.15 (0.08,0.30) | 0.21 (0.09,0.47) | 0.58 (0.32,1.07) | **UC** | 63.4 |
| **SUCRA** | 48.5 | 94.4 | 78.0 | 28.1 | 1.0 |  |
| **nCPAP** | **IVH** | | | | | **SUCRA** |
| **Death** | **InSurE** | 1.03 (0.63,1.67) | 0.92 (0.20,4.37) | 1.10 (0.25,4.71) | 0.62 (0.27,1.41) | 57.9 |
|  | 1.37 (0.92,2.04) | **LISA** | 0.90 (0.18,4.50) | 1.07 (0.24,4.72) | 0.60 (0.29,1.27) | 62.2 |
|  | 1.59 (0.25,10.31) | 1.16 (0.17,7.74) | **LMA** | 1.19 (0.14,9.79) | 0.67 (0.12,3.69) | 51.0 |
|  | 2.36 (0.56,9.97) | 1.72 (0.41,7.26) | 1.49 (0.15,15.20) | **SN** | 0.57 (0.13,2.52) | 59.4 |
|  | 1.19 (0.58,2.44) | 0.87 (0.45,1.67) | 0.75 (0.11,5.23) | 0.50 (0.13,1.98) | **UC** | 19.6 |
| **SUCRA** | 37.7 | 96.7 | 7.9 | 44.3 | 63.4 |  |
| **nCPAP** | **Adverse events** | | | | | **SUCRA** |
| **Air leak** | **InSurE** | 1.08 (0.49,2.37) | 1.33 (0.40,4.34) | 2.61 (0.26,26.35) | 1.25 (0.32,4.99) | 33.0 |
|  | 1.31 (0.76,2.24) | **LISA** | 1.23 (0.31,4.81) | 2.43 (0.27,21.92) | 1.17 (0.35,3.86) | 39.1 |
|  | 0.77 (0.36,1.61) | 0.59 (0.25,1.37) | **LMA** | 1.97 (0.16,23.57) | 0.95 (0.18,4.98) | 52.6 |
|  | 0.51 (0.21,1.26) | 0.39 (0.16,0.94) | 0.66 (0.24,1.86) | **SN** | 0.48 (0.08,3.05) | 77.1 |
|  | 0.54 (0.27,1.09) | 0.42 (0.22,0.79) | 0.71 (0.31,1.63) | 1.07 (0.57,2.02) | **UC** | 48.1 |
| **SUCRA** | 70.6 | 92.7 | 47.9 | 18.1 | 20.7 |  |
| **nCPAP** |  | | | | |  |
| **Secondary dose** | **InSurE** |  |  |  |  |  |
|  | 0.93 (0.65,1.33) | **LISA** |  |  |  |  |
|  | 1.01 (0.46,2.18) | 1.08 (0.48,2.45) | **LMA** |  |  |  |
|  | 0.54 (0.17,1.75) | 0.59 (0.17,2.02) | 0.54 (0.14,2.12) | **SN** |  |  |
|  | 3.24 (1.24,8.47) | 3.49 (1.35,9.03) | 3.22 (1.35,7.71) | 5.95 (1.41,25.11) | **UC** |  |
| **SUCRA** | 50.1 | 39.1 | 47.1 | 14.1 | 99.2 |  |

**G: The results of re-analysis excluding studies involving jet nebulizers.**

| **nCPAP** | **BPD** | | | | | **SUCRA** |
| --- | --- | --- | --- | --- | --- | --- |
| **Intubation** | **InSurE** | 1.66 (1.25,2.20) | 0.64 (0.31,1.33) | 1.06 (0.56,1.99) | 1.18 (0.71,1.96) | 39.7. |
|  | 1.90 (1.37,2.64) | **LISA** | 0.39 (0.18,0.83) | 0.64 (0.35,1.17) | 0.71 (0.45,1.13) | 96.0 |
|  | 1.63 (0.80,3.32) | 0.86 (0.40,1.83) | **LMA** | 1.66 (0.68,4.02) | 1.84 (0.83,4.09) | 8.2 |
|  | 0.62 (0.26,1.46) | 0.32 (0.14,0.77) | 0.38 (0.14,1.02) | **SN** | 1.11 (0.73,1.70) | 45.8 |
|  | 0.32 (0.16,0.62) | 0.17 (0.09,0.33) | 0.20 (0.09,0.43) | 0.51 (0.27,0.97) | **UC** | 60.4 |
| **SUCRA** | 48.7 | 91.1 | 80.9 | 28.8 | 0.5 |  |
| **nCPAP** |  | | | | |  |
| **Air leak** | **InSurE** |  |  |  |  |  |
|  | 1.35 (0.82,2.23) | **LISA** |  |  |  |  |
|  | 0.77 (0.37,1.62) | 0.57 (0.25,1.32) | **LMA** |  |  |  |
|  | 0.51 (0.20,1.30) | 0.38 (0.15,0.94) | 0.66 (0.23,1.91) | **SN** |  |  |
|  | 0.56 (0.28,1.10) | 0.41 (0.22,0.77) | 0.72 (0.31,1.65) | 1.08 (0.54,2.17) | **UC** |  |
| **SUCRA** | 69.0 | 94.1 | 47.4 | 18.4 | 21.1 |  |

**H: The results of re-analysis excluding the study by Sadeghnia, A. R., 2022 for IVH outcomes.**

| **nCPAP** |  | | | | |  |
| --- | --- | --- | --- | --- | --- | --- |
|  | **InSurE** |  |  |  |  |  |
|  | 1.01 (0.68,1.50) | **LISA** |  |  |  |  |
|  | 0.90 (0.20,4.09) | 0.90 (0.19,4.23) | **LMA** |  |  |  |
|  | 0.29 (0.03,2.62) | 0.29 (0.03,2.54) | 0.32 (0.02,4.48) | **SN** |  |  |
|  | 0.53 (0.25,1.13) | 0.52 (0.26,1.04) | 0.58 (0.11,3.01) | 1.83 (0.23,14.50) | **UC** |  |
| **SUCRA** | 71.7 | 72.3 | 61.3 | 18.6 | 26.1 |  |

Abbreviations: InSurE, Intubate, Surfactant, Extubate; LISA, less invasive surfactant administration; LMA, laryngeal mask airway; SN, surfactant nebulization; UC,usual care; SUCRA, the surface under the cumulative ranking curve.

**Supplementary Table 4: Results of Loop-specific approach.**

| **LOOP** | **ROR** | **z_value** | **p_value** | **CI_95** | **Loop_Heterog_tau^2^** |
| --- | --- | --- | --- | --- | --- |
| Intubation | | | | | |
| INSURE-SN-UC | 14.975 | 1.608 | 0.108 | (1.00,405.61) | 0.776 |
| INSURE-LMA-UC | 4.537 | 1.091 | 0.275 | (1.00,68.69) | 0.587 |
| INSURE-LISA-UC | 1.296 | 0.312 | 0.755 | (1.00,6.60) | 0.066 |
| BronchopulmonaryDysplasia | | | | | |
| INSURE-SN-UC | 15.614 | 1.550 | 0.121 | (1.00,504.67) | 0.000 |
| INSURE-LMA-UC | 8.604 | 1.220 | 0.223 | (1.00,273.48) | 0.000 |
| INSURE-LISA-UC | 5.929 | 1.106 | 0.269 | (1.00,138.74) | 0.000 |
| Death | | | | | |
| INSURE-LMA-UC | 4.469 | 0.620 | 0.535 | (1.00,506.11) | 0.000 |
| INSURE-LISA-UC | 3.607 | 1.440 | 0.150 | (1.00,20.67) | 0.000 |
| INSURE-SN-UC | 2.396 | 0.514 | 0.607 | (1.00,66.90) | 0.000 |
| IVH | | | | | |
| INSURE-SN-UC | 70.833 | 2.662 | 0.008 | (3.08,1630.90) | 0.000 |
| INSURE-LMA-UC | 7.548 | 0.869 | 0.385 | (1.00,721.34) | 0.000 |
| INSURE-LISA-UC | 2.930 | 1.113 | 0.266 | (1.00,19.45) | 0.000 |
| Airleak | | | | | |
| INSURE-SN-UC | 5.917 | 1.167 | 0.243 | (1.00,117.06) | 0.000 |
| INSURE-LISA-UC | 1.302 | 0.285 | 0.775 | (1.00,7.96) | 0.000 |
| INSURE-LMA-UC | 1.236 | 0.200 | 0.842 | (1.00,9.91) | 0.000 |
| NEC | | | | | |
| - | - | - | - | - | - |
| PDA | | | | | |
| INSURE-LISA-UC | 12.307 | 1.864 | 0.062 | (1.00,172.45) | 0.000 |
| ROP | | | | | |
| - | - | - | - | - | - |
| Adverse events during surfactant administration | | | | | |
| INSURE-LISA-LMA-UC | 1.968 | 0.355 | 0.722 | (1.00,82.15) | 0.664 |
| Secondary dose administration | | | | | |
| INSURE-LISA-SN-UC | 31.626 | 2.541 | 0.011 | (2.20,454.22) | 0.000 |
| INSURE-LMA-SN-UC | 23.792 | 1.361 | 0.174 | (1.00,2285.84) | 1.262 |
| INSURE-LISA-LMA-UC | 1.303 | 0.290 | 0.771 | (1.00,7.79) | 0.000 |
| Duration of mechanical ventilation | | | | | |
| - | - | - | - | - | - |

Abbreviations: InSurE, Intubate, Surfactant, Extubate; LISA, less invasive surfactant administration; LMA, laryngeal mask airway; SN, surfactant nebulization; UC,usual care; BPD, Bronchopulmonary dysplasia; IVH, Severe intraventricular hemorrhage; ROP, Retinopathy of prematurity; NEC, Neonatal necrotizing enterocolitis; PDA, Patent ductus arteriosus; CI, confidence interval.

**Supplementary Table 5: Results of Side-splitting approach.**

| **Side** | **Direct** | | **Indirect** | | **Difference** | | | **tau** |
| --- | --- | --- | --- | --- | --- | --- | --- | --- |
|  | **Coef.** | **Std.Err.** | **Coef.** | **Std.Err.** | **Coef.** | **Std.Err.** | **P>\|z\|** |  |
| **Intubation** | | | | | | | | |
| InSurE LISA | -0.566 | 0.174 | -1.752 | 0.663 | 1.186 | 0.686 | 0.084 | 0.4819 |
| InSurE LMA | -0.783 | 0.426 | 0.257 | 0.683 | -1.040 | 0.805 | 0.196 | 0.4741 |
| InSurE SN | -1.048 | 0.898 | 1.042 | 0.500 | -2.090 | 1.028 | 0.042 | 0.4788 |
| InSurE UC | 1.856 | 0.845 | 0.992 | 0.373 | 0.864 | 0.924 | 0.349 | 0.4869 |
| LISA UC | 2.310 | 0.463 | 1.124 | 0.504 | 1.186 | 0.686 | 0.084 | 0.4819 |
| LMA UC | 1.110 | 0.566 | 2.150 | 0.573 | -1.040 | 0.805 | 0.196 | 0.4741 |
| SN UC | 0.355 | 0.327 | 2.444 | 0.970 | -2.090 | 1.028 | 0.042 | 0.4788 |
| **BPD** | | | | | | | | |
| InSurE LISA | -0.482 | 0.149 | -0.871 | 0.590 | 0.389 | 0.608 | 0.522 | 0.0000 |
| InSurE LMA | 0.357 | 0.444 | 0.670 | 0.704 | -0.313 | 0.833 | 0.707 | 0.0000 |
| InSurE SN | -0.840 | 0.773 | 0.137 | 0.353 | -0.977 | 0.850 | 0.251 | 0.0000 |
| InSurE UC | 1.707 | 1.580 | -0.219 | 0.264 | 1.927 | 1.602 | 0.229 | 0.0000 |
| LISA UC | 0.409 | 0.260 | 0.020 | 0.550 | 0.389 | 0.608 | 0.522 | 0.0000 |
| LMA UC | -0.802 | 0.648 | -0.490 | 0.523 | -0.313 | 0.833 | 0.707 | 0.0000 |
| SN UC | -0.201 | 0.221 | 0.775 | 0.821 | -0.977 | 0.850 | 0.251 | 0.0000 |
| **Death** | | | | | | | | |
| InSurE LISA | -0.304 | 0.162 | 0.678 | 0.769 | -0.982 | 0.786 | 0.211 | 0.0000 |
| InSurE LMA | -0.564 | 1.078 | -0.066 | 2.041 | -0.498 | 2.308 | 0.829 | 0.0000 |
| InSurE SN | -0.736 | 1.259 | -0.880 | 0.899 | 0.145 | 1.547 | 0.926 | 0.0000 |
| InSurE UC | 0.875 | 0.789 | -0.386 | 0.393 | 1.261 | 0.881 | 0.152 | 0.0000 |
| LISA UC | -0.103 | 0.380 | 0.879 | 0.687 | -0.982 | 0.786 | 0.211 | 0.0000 |
| LMA UC | -0.058 | 2.010 | 0.441 | 1.135 | -0.498 | 2.308 | 0.829 | 0.0000 |
| SN UC | 0.737 | 0.824 | 0.593 | 1.310 | 0.145 | 1.547 | 0.926 | 0.0000 |
| **IVH** | | | | | | | | |
| InSurE LISA | -0.084 | 0.226 | 0.169 | 0.853 | -0.253 | 0.882 | 0.774 | 0.3772 |
| InSurE LMA | 0.000 | 0.847 | 0.480 | 2.080 | -0.480 | 2.246 | 0.831 | 0.3523 |
| InSurE SN | -1.689 | 0.901 | 1.244 | 1.127 | -2.933 | 1.442 | 0.042 | 0.2899 |
| InSurE UC | 1.964 | 0.934 | 0.069 | 0.463 | 1.894 | 1.042 | 0.069 | 0.3528 |
| LISA UC | 0.409 | 0.429 | 0.662 | 0.774 | -0.253 | 0.882 | 0.774 | 0.3772 |
| LMA UC | -0.058 | 2.040 | 0.423 | 0.940 | -0.480 | 2.246 | 0.831 | 0.3523 |
| SN UC | -0.603 | 1.057 | 2.330 | 0.981 | -2.933 | 1.442 | 0.042 | 0.2899 |
| **Airleak** | | | | | | | | |
| InSurE LISA | -0.294 | 0.280 | -0.381 | 0.627 | 0.088 | 0.686 | 0.898 | 0.0000 |
| InSurE LMA | 0.213 | 0.366 | 0.090 | 0.725 | 0.124 | 0.812 | 0.879 | 0.0000 |
| InSurE SN | -0.736 | 1.259 | 0.845 | 0.488 | -1.581 | 1.350 | 0.242 | 0.0000 |
| InSurE UC | 0.875 | 0.789 | 0.497 | 0.381 | 0.378 | 0.876 | 0.666 | 0.0000 |
| LISA UC | 0.905 | 0.392 | 0.818 | 0.564 | 0.088 | 0.686 | 0.898 | 0.0000 |
| LMA UC | 0.450 | 0.610 | 0.327 | 0.536 | 0.124 | 0.812 | 0.879 | 0.0000 |
| SN UC | -0.167 | 0.335 | 1.414 | 1.308 | -1.581 | 1.350 | 0.242 | 0.0000 |
| **NEC** | | | | | | | | |
| InSurE LISA* | -0.323 | 0.325 | 0.035 | 622.254 | -0.358 | 622.254 | 1.000 | 0.0000 |
| InSurE LMA | . | . | . | . | . | . | . | . |
| LISA UC* | -0.034 | 0.331 | 0.434 | 812.441 | -0.468 | 812.442 | 1.000 | 0.0000 |
| SN UC* | -0.010 | 0.750 | -0.782 | 1887.350 | 0.772 | 1887.350 | 1.000 | 0.0000 |
| **PDA** | | | | | | | | |
| InSurE LMA | -0.313 | 0.146 | 2.164 | 1.358 | -2.478 | 1.366 | 0.070 | 0.1655 |
| InSurE SN | . | . | . | . | . | . | . | . |
| InSurE UC | 3.283 | 1.128 | 0.806 | 0.771 | 2.478 | 1.366 | 0.070 | 0.1655 |
| LISA UC | 1.119 | 0.756 | 3.597 | 1.137 | -2.478 | 1.366 | 0.070 | 0.1655 |
| SN UC* | 0.280 | 0.460 | 3.107 | 1195.809 | -2.827 | 1195.809 | 0.998 | 0.1903 |
| **ROP** | | | | | | | | |
| InSurE LISA* | -0.143 | 0.281 | -0.422 | 809.011 | 0.280 | 809.011 | 1.000 | 0.0000 |
| InSurE LMA | . | . | . | . | . | . | . | . |
| LISA UC* | 0.514 | 0.667 | 0.135 | 954.957 | 0.379 | 954.957 | 1.000 | 0.0000 |
| SN UC* | -0.376 | 1.061 | 0.532 | 2135.277 | -0.908 | 2135.277 | 1.000 | 0.0000 |
| **Adverse events during surfactant administration** | | | | | | | | |
| InSurE LISA | -0.162 | 0.311 | 0.518 | 1.689 | -0.680 | 1.717 | 0.692 | 0.8323 |
| InSurE LMA | -0.381 | 0.537 | -1.060 | 1.631 | 0.680 | 1.717 | 0.692 | 0.8323 |
| LISA UC | -0.246 | 0.576 | 0.434 | 1.617 | -0.680 | 1.717 | 0.692 | 0.8323 |
| LMA UC | 0.653 | 1.494 | -0.027 | 0.846 | 0.680 | 1.717 | 0.692 | 0.8323 |
| SN UC* | 0.781 | 0.851 | -0.697 | 1774.256 | 1.478 | 1774.256 | 0.999 | 0.8053 |
| **Secondary dose administration** | | | | | | | | |
| InSurE LISA | 0.097 | 0.151 | -0.861 | 0.912 | 0.958 | 0.916 | 0.295 | 0.1156 |
| InSurE LMA | 0.150 | 0.409 | -0.450 | 0.767 | 0.599 | 0.860 | 0.486 | 0.0648 |
| InSurE SN | -0.170 | 0.583 | 3.137 | 1.119 | -3.307 | 1.262 | 0.009 | 0.0000 |
| LISA UC | -0.652 | 0.674 | -1.611 | 0.621 | 0.958 | 0.916 | 0.295 | 0.1155 |
| LMA UC | -0.969 | 0.461 | -1.568 | 0.726 | 0.599 | 0.860 | 0.486 | 0.0648 |
| SN UC | -3.837 | 1.023 | -0.530 | 0.739 | -3.307 | 1.262 | 0.009 | 0.0000 |
| **Duration of mechanical ventilation** | | | | | | | | |
| InSurE LISA* | -0.751 | 0.538 | -0.346 | 210.645 | -0.405 | 210.646 | 0.998 | 1.4098 |
| LISA UC* | 0.484 | 0.943 | 0.930 | 269.333 | -0.447 | 269.335 | 0.999 | 1.4098 |
| LMA UC* | 0.125 | 1.423 | -0.531 | 616.377 | 0.656 | 616.378 | 0.999 | 1.4098 |
| SN UC* | -0.493 | 1.688 | 0.516 | 3639.778 | -1.010 | 3639.777 | 1.000 | 1.4098 |

Abbreviations: InSurE, Intubate, Surfactant, Extubate; LISA, less invasive surfactant administration; LMA, laryngeal mask airway; SN, surfactant nebulization; UC,usual care; BPD, Bronchopulmonary dysplasia; IVH, Severe intraventricular hemorrhage; ROP, Retinopathy of prematurity; NEC, Neonatal necrotizing enterocolitis; PDA, Patent ductus arteriosus.

**Supplementary Fig 1: Plots of the surface under the cumulative ranking curves for all interventions.**

1. **Intubation**


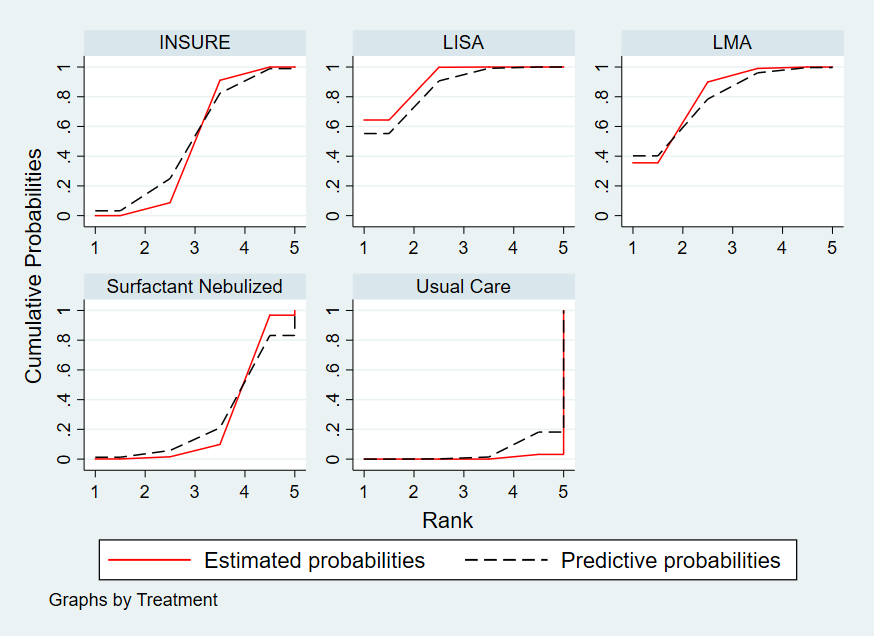


1. **BPD**


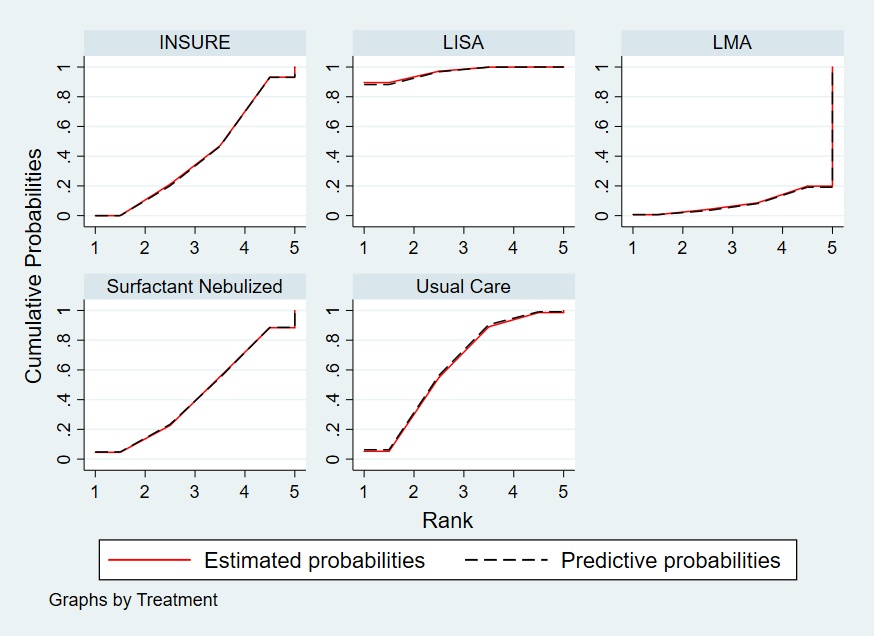


1. **Death**


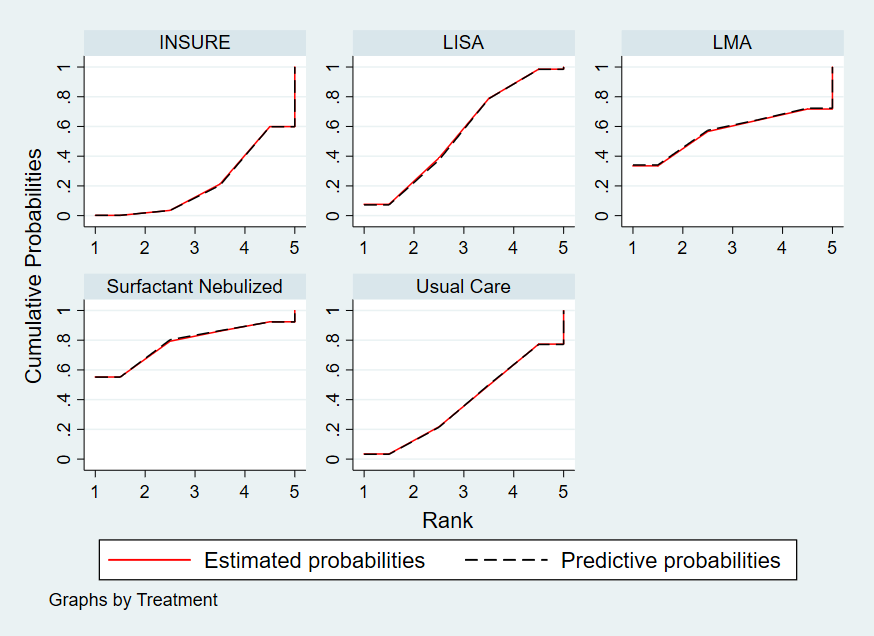


1. **IVH**


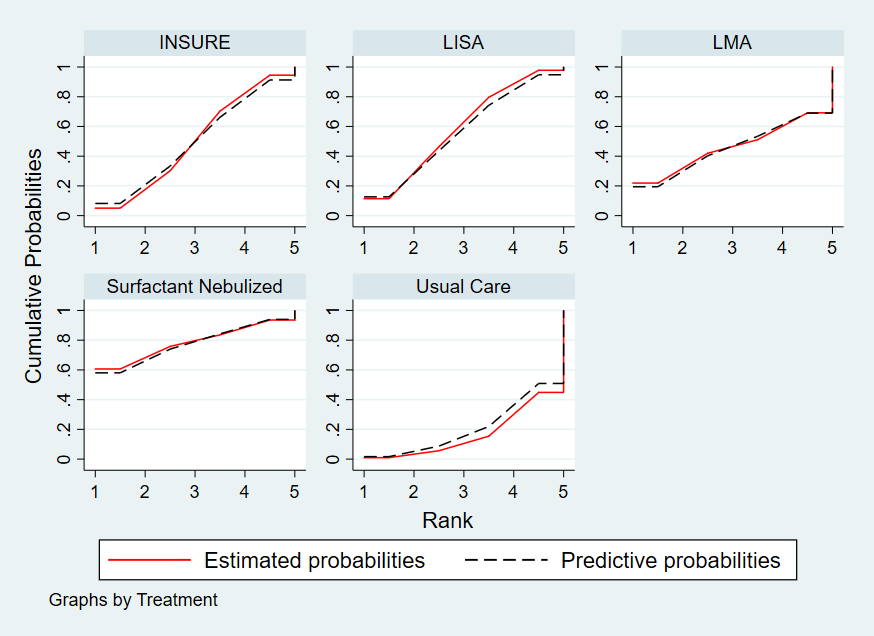


1. **Air leak**


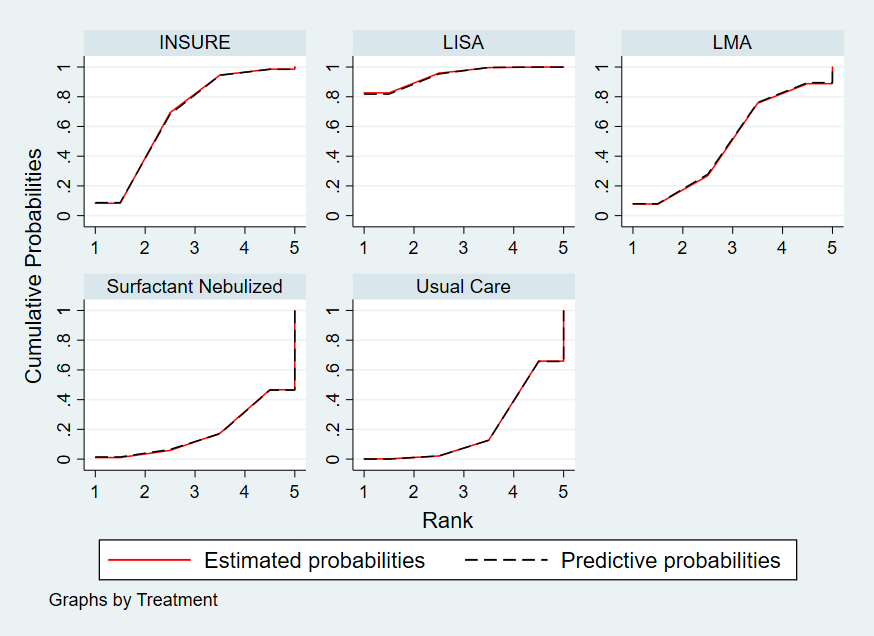


1. **NEC**


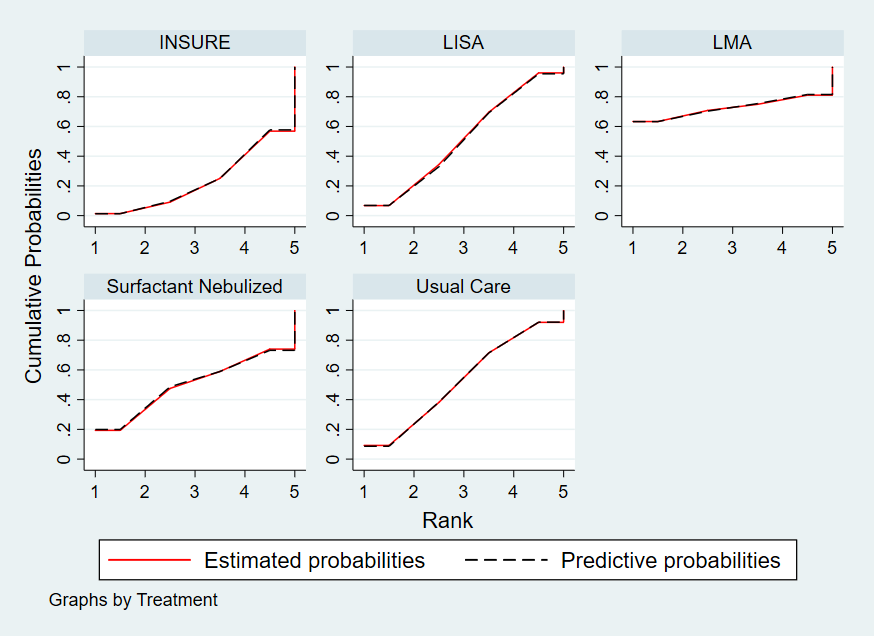


1. **PDA**


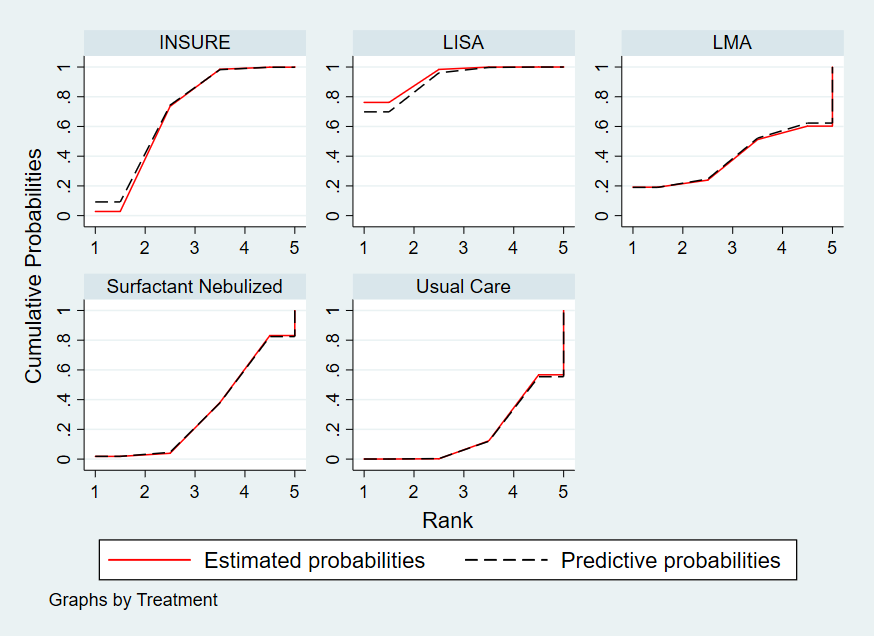


1. **ROP**


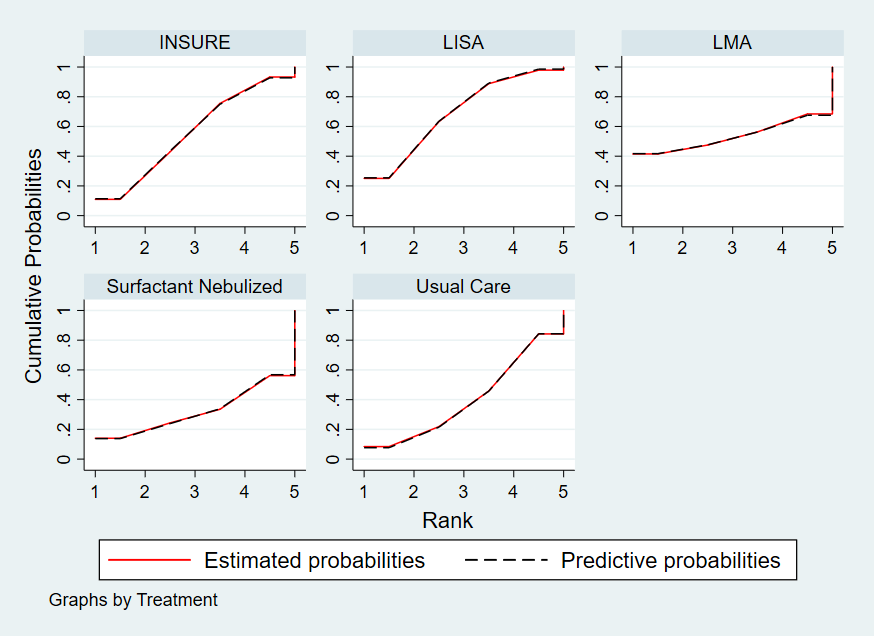


1. **Adverse events during surfactant administration**


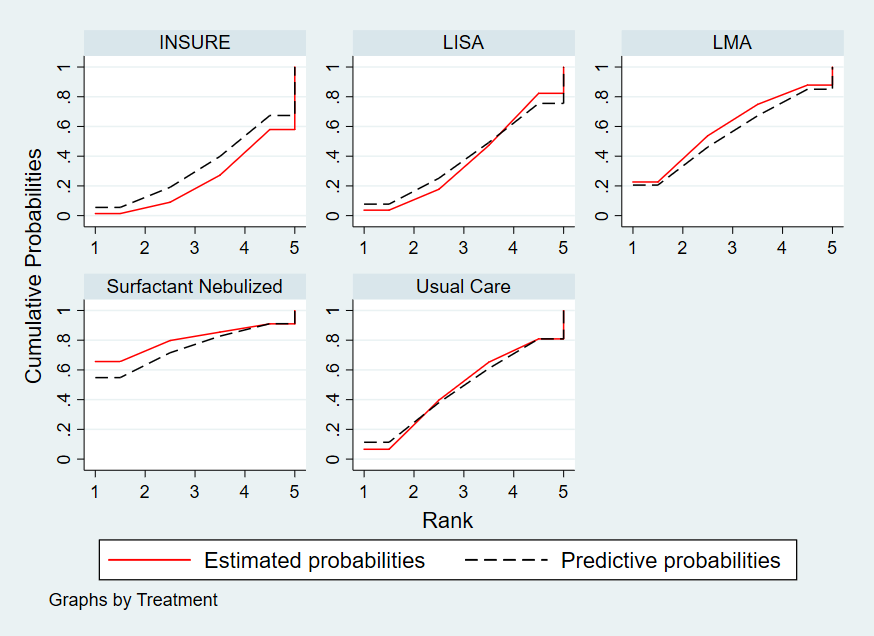


1. Secondary dose administration


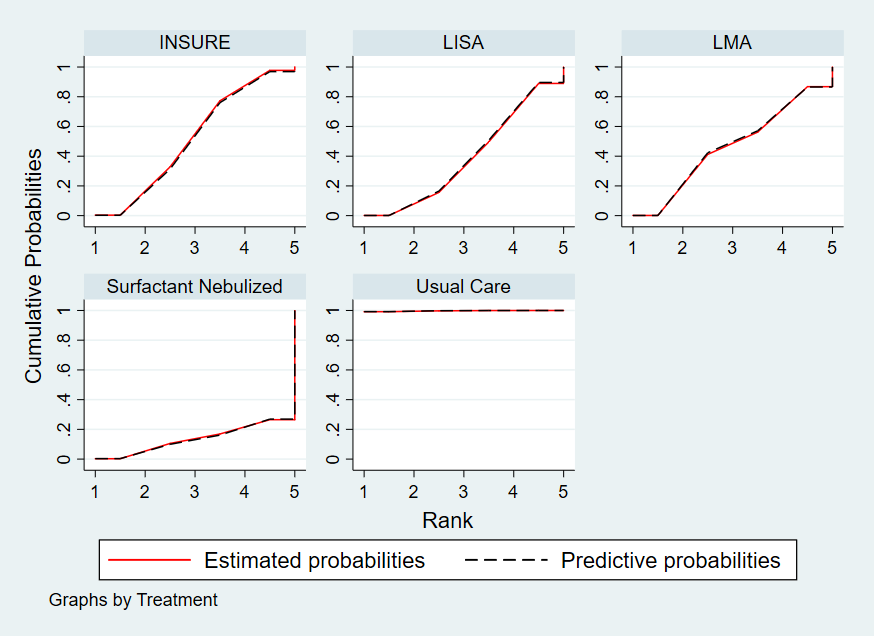


1. **Duration of mechanical ventilation**


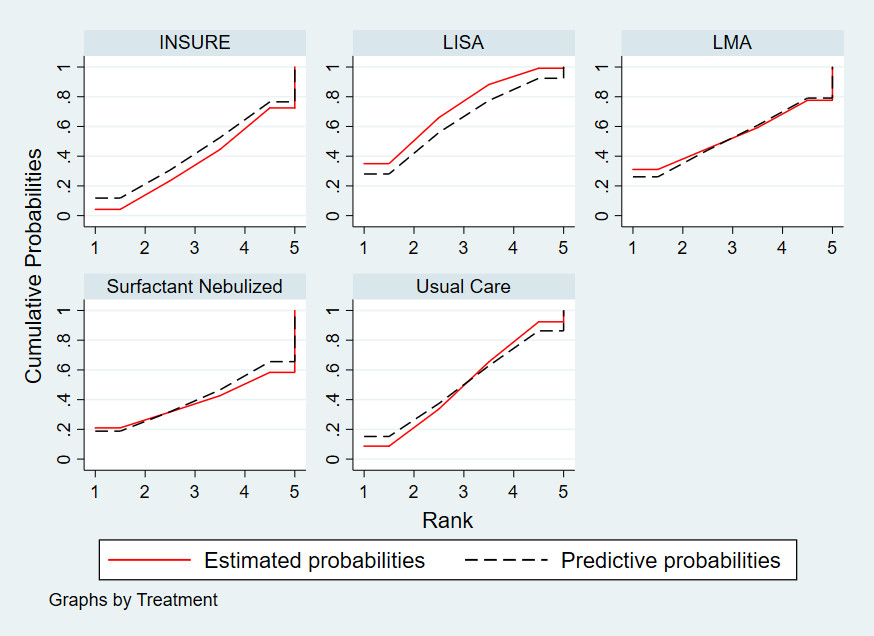


**Supplementary Fig 2: Comparison-adjusted funnel plot with pseudo 95% confidence limits.**

1. **Intubation**


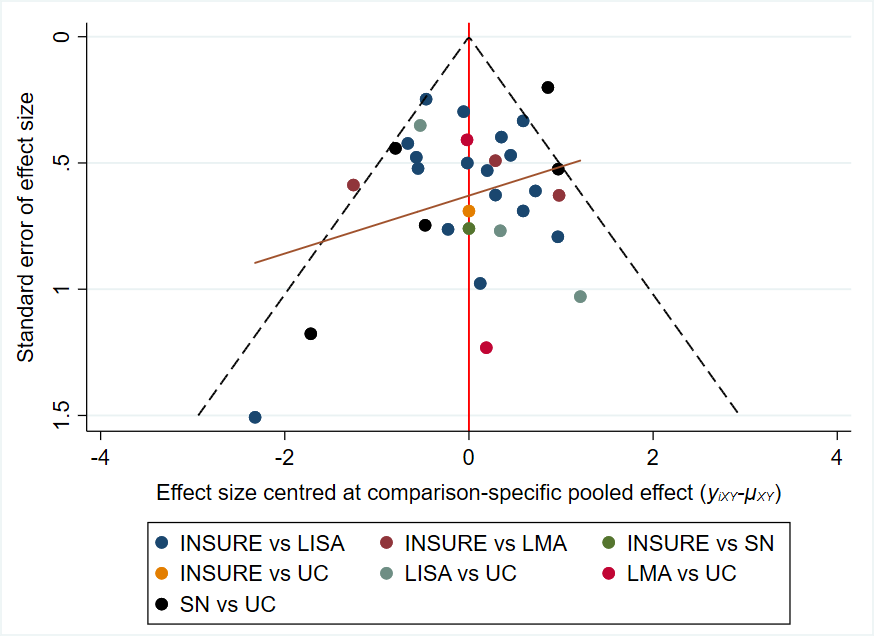


1. **BPD**


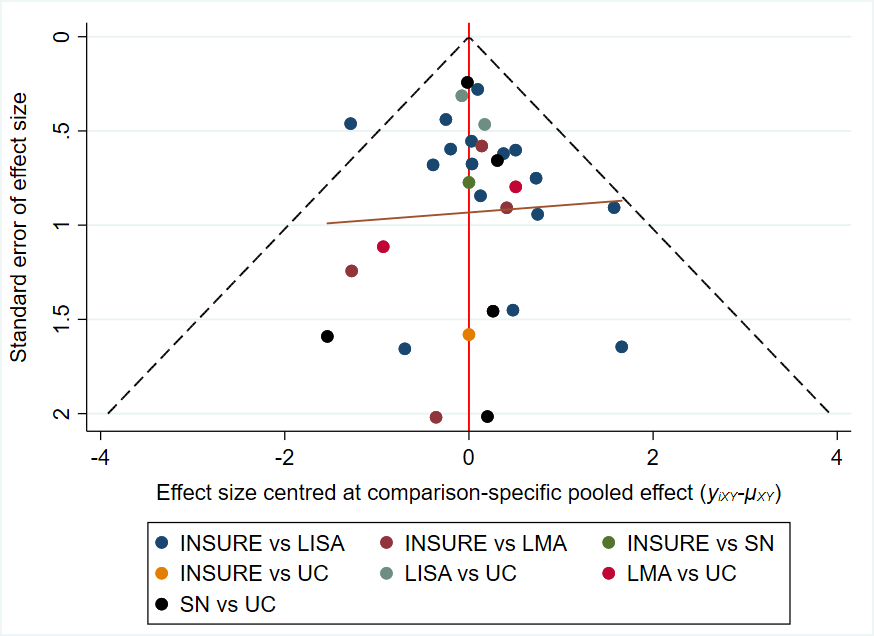


1. **Death**


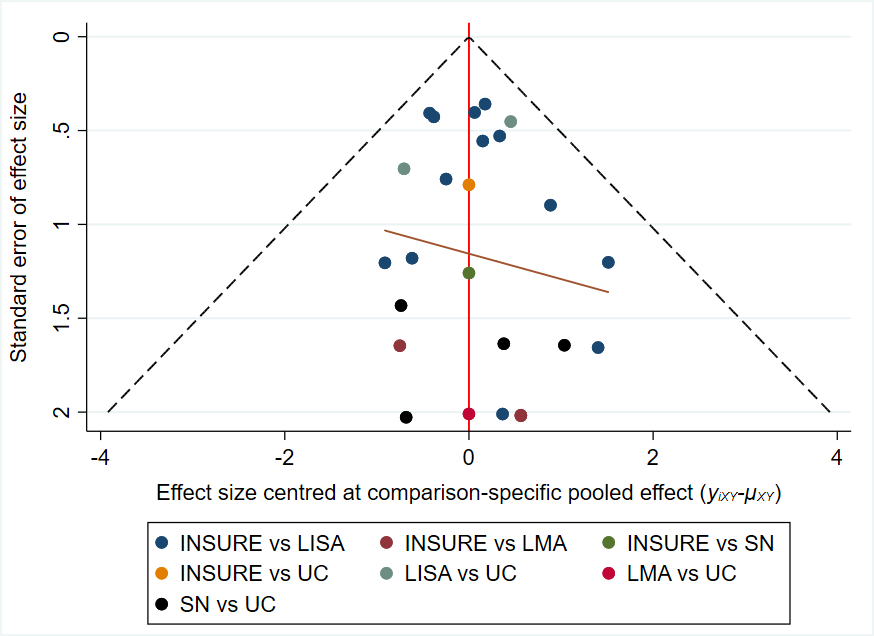


1. **IVH**


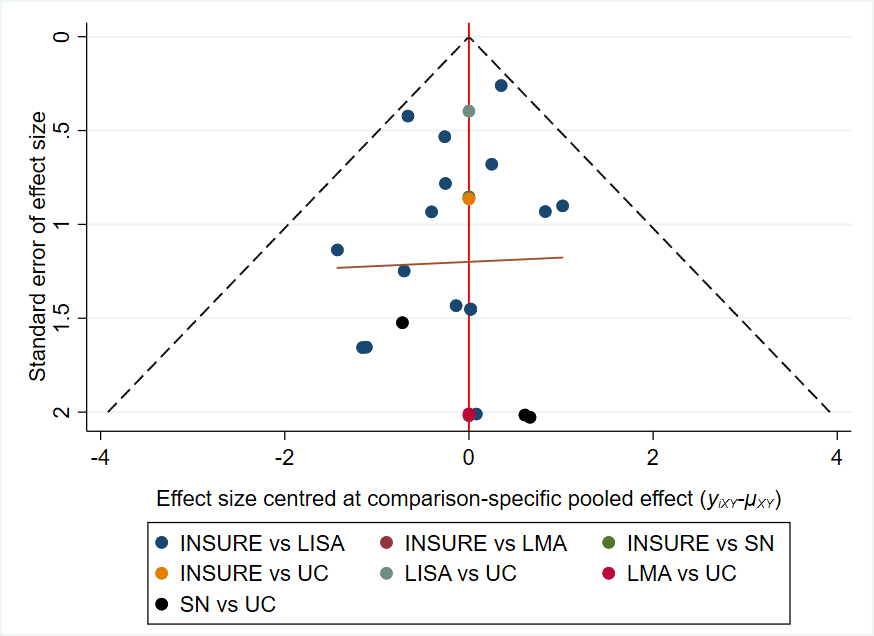


1. **Air leak**


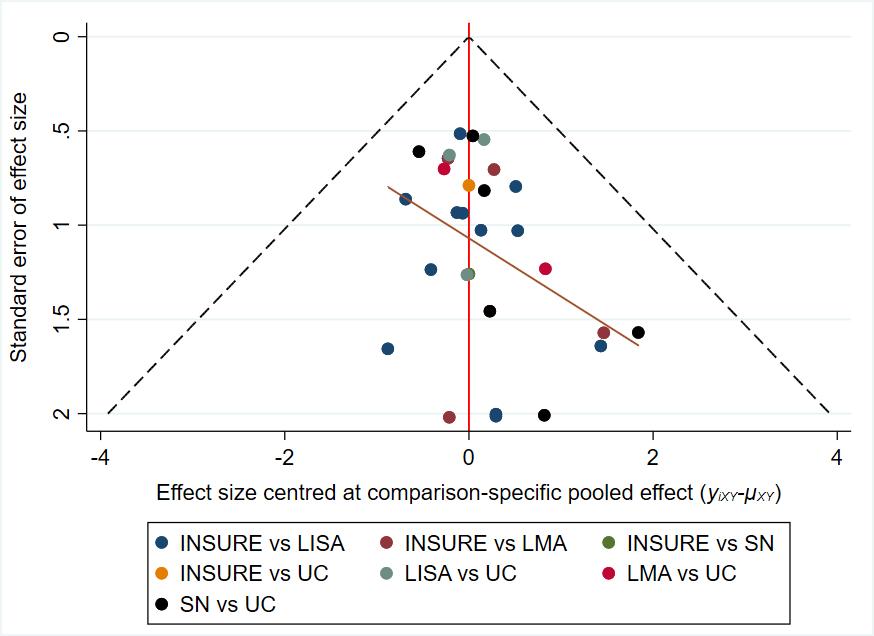


1. **NEC**


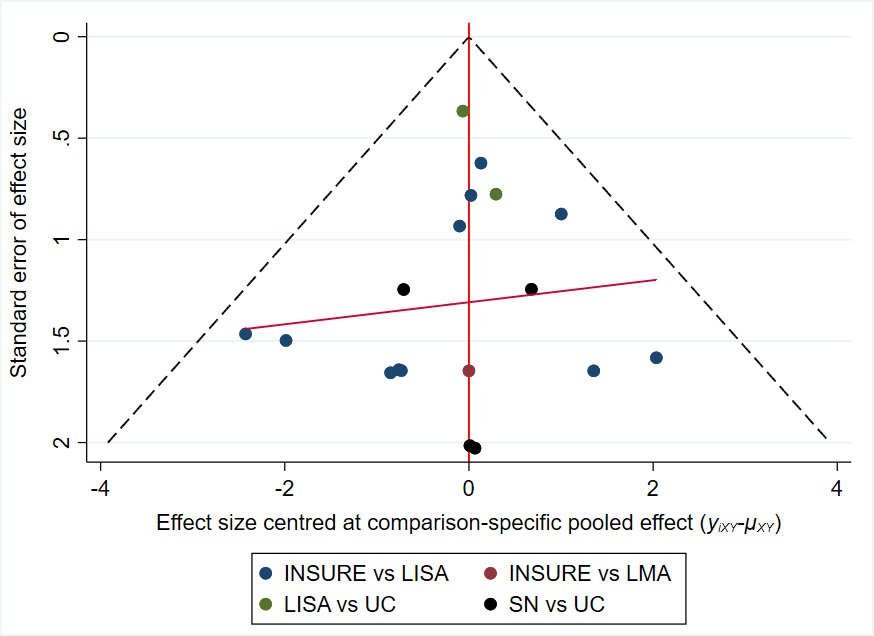


1. **PDA**


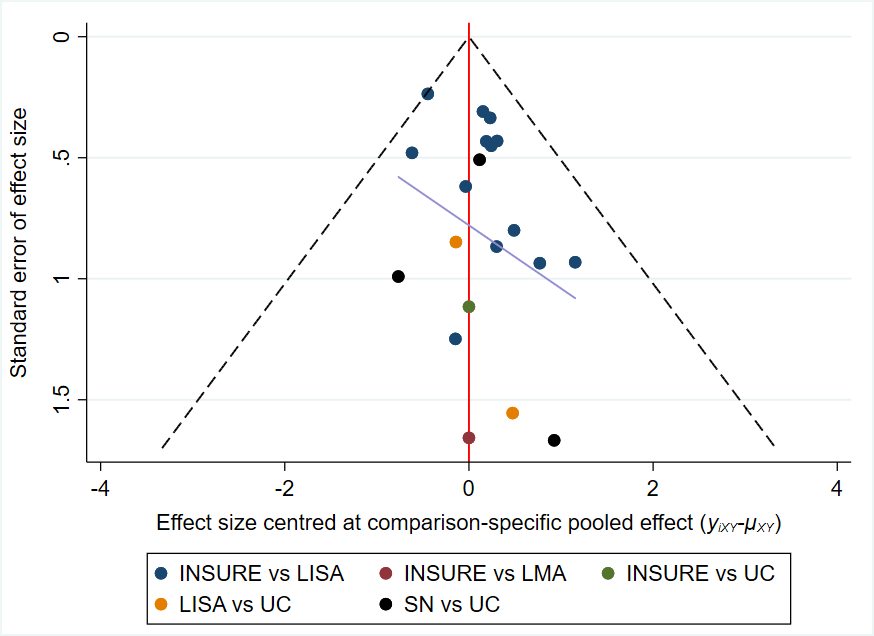


1. **ROP**


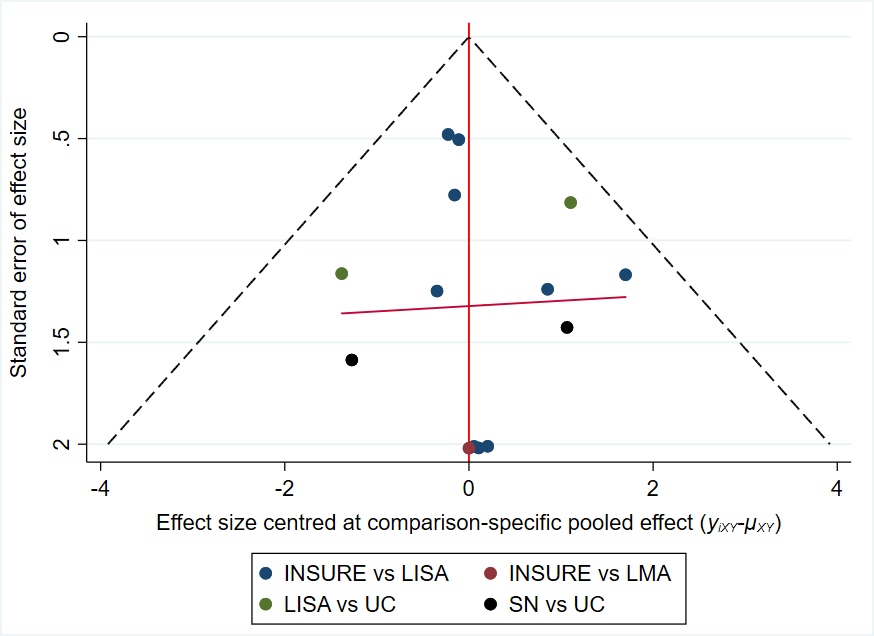


1. **Adverse events during surfactant administration**


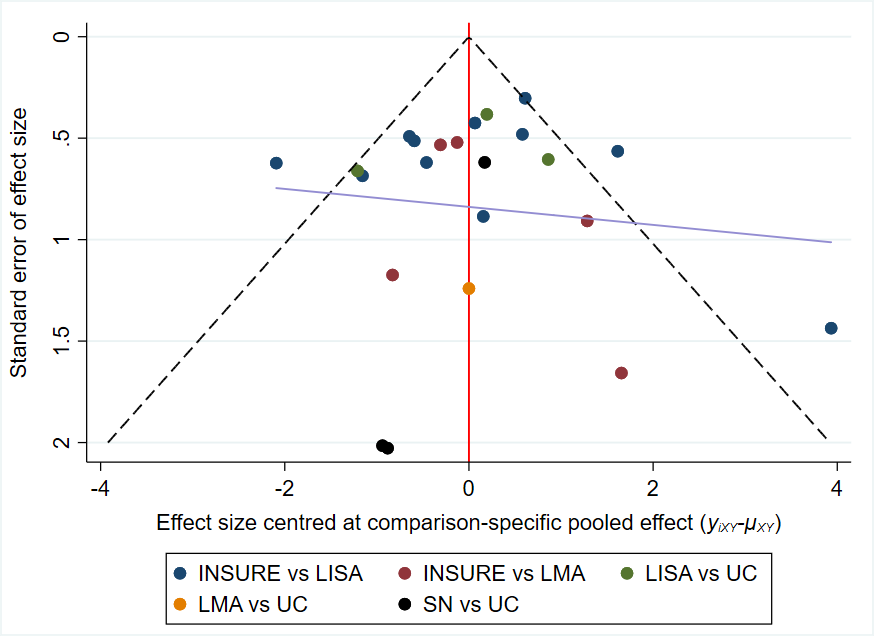


1. **Secondary dose administration**


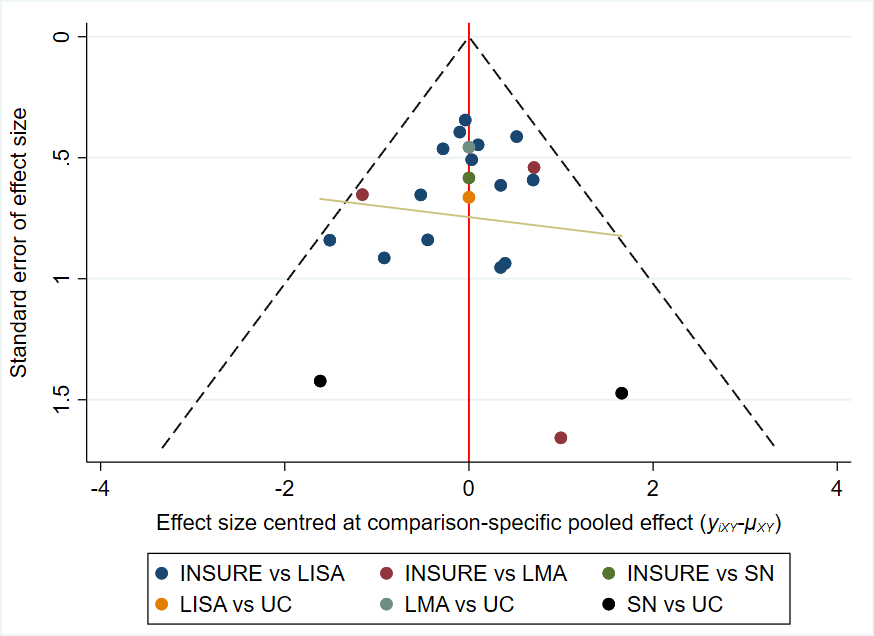


1. **Duration of mechanical ventilation**


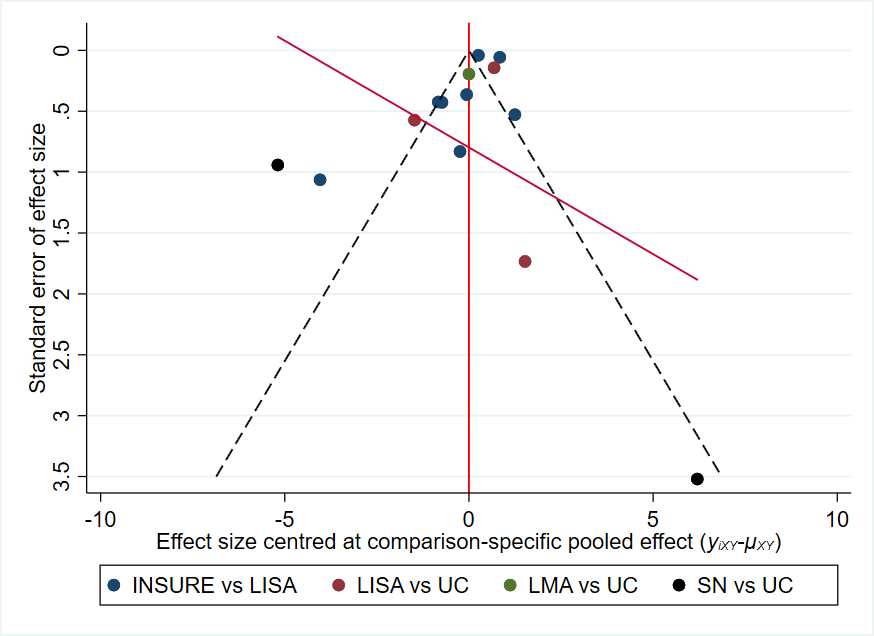


Abbreviations: InSurE, Intubate, Surfactant, Extubate; LISA, less invasive surfactant administration; LMA, laryngeal mask airway; SN, surfactant nebulization; UC,usual care; BPD, Bronchopulmonary dysplasia; IVH, Severe intraventricular hemorrhage; ROP, Retinopathy of prematurity; NEC, Neonatal necrotizing enterocolitis; PDA, Patent ductus arteriosus.
