# Supplementary material for: Comparative efficacy and safety of pulmonary surfactant delivery strategies in neonatal RDS: a network meta-analysis
Source: BMC Pulm Med. 2024 Dec 30;24:637. doi: 10.1186/s12890-024-03429-4 (PMC11687079; doi:10.1186/s12890-024-03429-4)
Supplement: Supplementary file 2 — Supplementary Material 2. Appendix 1: Changes of the final review protocol from the original one in PROSPERO and reasons. [file 12890_2024_3429_MOESM2_ESM.doc]

**Appendix 1: Changes of the final review protocol from the original one in PROSPERO and reasons.**

| **Sections** | **Original protocol (PROSPERO)** | **Final systematic review and manuscript** | **Reasons for the changes** |
| --- | --- | --- | --- |
| **Date of last search** | We would systematically review the databases by July 2023. | We conducted a systematic search of databases up to November 2023. | Delay in developing search strategies. |
| **Intervention(s)** | Eligible interventions were 4 interventions not including  usual care and laryngeal mask airway. And the LISA and MIST methods are two separate interventions. | This review included 5 interventions, including usual care and laryngeal mask airway. And all methods of PS administration via catheter have been combined into one single node named LISA (less invasive surfactant administration) or transcatheter administration, including the Cologne Method (LISA) and the Hobart Method (MIST), and extensions based on these two methods. | Some trials included based on the original protocol compared the four interventions with usual care, necessitating the inclusion of the usual care strategy for a comprehensive indirect comparison among interventions. The potential of the LMA strategy is noteworthy and has been included. During data extraction, similarities in the definitions of the LISA and MIST methods were identified, and the boundaries between the two were unclear in the articles. Consequently, we deemed it appropriate to combine the LISA and MIST methods in the original protocol. |
| **Secondary Outcome(s)** | The PROSPERO protocol  included SpO2, PaCO2, FiO2, heart rate, blood pressure, mean assisted ventilation duration, Retinopathy of Prematurity (ROP), neonatal necrotizing enterocolitis (NEC), patent ductus arteriosus (PDA), and neurodevelopment. | This review included severe intraventricular hemorrhage (IVH), retinopathy of prematurity (ROP), neonatal necrotizing enterocolitis (NEC), patent ductus arteriosus (PDA), incidences of air leak, secondary dose administration, adverse events during surfactant administration, duration of mechanical ventilation, duration of oxygen support and length of hospital stay. | Due to the limited availability of articles reporting indicators such as SpO2, PaCO2, FiO2, heart rate, and blood pressure, we refrained from analyzing them as secondary endpoints. |
| **Demographics of included studies** | The demographics of The PROSPERO protocol about included trails embraced study design, sample size, gestational age (wk), birth weight (g), maternal age (yr), APGAR score, cesarean section, and maternal disease. | This review included study design, sample size, gestational age (wk), birth weight (g). | Data on prenatal and perinatal conditions were difficult to collect due to the limited content of the article reports. |
| **Sensitivity analysis** | No subgroup analysis | This review conducted subgroup analyses based on various factors, including nebulizer types, age, medication dosage, and primary mode of respiratory support. | This is because of potential heterogeneity in baseline levels across intervention groups. |
